# Identification and Characterization of LINE and SINE Retrotransposons in the African Hedgehog (*Atelerix albiventris*, *Erinaceidae*) and Their Association with 3D Genome Organization and Gene Expression

**DOI:** 10.3390/genes16040397

**Published:** 2025-03-29

**Authors:** Mengyuan Zhu, Jianxuan Zhou, Nannan Chen, Jianing Xu, Haipeng Wang, Libo Jiang, Fengtang Yang

**Affiliations:** 1 School of Computer Science and Technology, Shandong University of Technology, Zibo 255000, China; 2 School of Life Sciences and Medicine, Shandong University of Technology, Zibo 255000, Chinaxujianing1219@126.com (J.X.)

**Keywords:** repetitive sequences, LINEs (long interspersed nuclear elements), SINEs (short interspersed nuclear elements), *Atelerix albiventris*, genome structure

## Abstract

Background: The African hedgehog (*Atelerix albiventris*) exhibits specialized skin differentiation leading to spine formation, yet its regulatory mechanisms remain unclear. Transposable elements (TEs), particularly LINEs (long interspersed nuclear elements) and SINEs (short interspersed nuclear elements), are known to influence genome organization and gene regulation. Objectives: Given the high proportion of SINEs in the hedgehog genome, this study aims to characterize the distribution, evolutionary dynamics, and potential regulatory roles of LINEs and SINEs, focusing on their associations with chromatin architecture, DNA methylation, and gene expression. Methods: We analyzed LINE and SINE distribution using HiFi sequencing and classified TE families through phylogenetic reconstruction. Hi-C data were used to explore TE interactions with chromatin architecture, while whole-genome 5mCpG methylation was inferred from PacBio HiFi reads of muscle tissue using a deep-learning-based approach. RNA-seq data from skin tissues were analyzed to assess TE expression and potential associations with genes linked to spine development. Results: SINEs form distinct genomic blocks in GC-rich and highly methylated regions, whereas LINEs are enriched in AT-rich, hypomethylated regions. LINEs and SINEs are associated differently with A/B compartments, with SINEs in euchromatin and LINEs in heterochromatin. Methylation analysis suggests that younger TEs tend to have higher methylation levels, and expression analysis indicates that some differentially expressed TEs may be linked to genes involved in epidermal and skeletal development. Conclusions: This study provides a genome-wide perspective on LINE and SINE distribution, methylation patterns, and potential regulatory roles in *A. albiventris*. While not establishing a direct causal link, the findings suggest that TEs may influence gene expression associated with spine development, offering a basis for future functional studies.

## 1. Introduction

Hedgehogs, a unique group of nocturnal mammals covered in protective spines, use these spines as a formidable defensive mechanism. The four-toed hedgehog (*A. albiventris*) is one of four members of the *Atelerix* genus within the family Erinaceidae. It is the smallest African hedgehog species and has been domesticated for biomedical research and as a pet [1]. Like most hedgehogs, *A. albiventris* has 48 chromosomes (2*n* = 48). Cytogenetic studies have shown that large AT-enriched heterochromatin fragments are located on the long arms of three autosomes in the *A. albiventris* genome [2]. Two hedgehog genomes, *Erinaceus europaeus* and *A. albiventris*, have been sequenced and are publicly available [3]. The initial analysis of repetitive sequences in these genomes revealed unique features, notably the significant expansion of SINE families compared to other mammals [3]. A similar trend has been observed in the rabbit genome, where 19.61% of TEs are SINEs—the highest proportion among studied species [4]. Cytogenetic and genomic studies suggest that these unique features, such as heterochromatin formation and SINE expansion, are closely linked to TE regulation, further supporting the idea that TEs play a crucial role in genome evolution, spine development, and higher-order genome structure in hedgehogs.

Transposable elements (TEs) are mobile genetic elements that move throughout the host genome, affecting both euchromatin and heterochromatin regions. TEs contribute significantly to genome size by inserting new copies at various positions. For instance, TEs comprise 45% of the mouse genome, 50–70% of the human genome, and 90% of the wheat genome [5,6,7]. TEs can regulate neighboring gene expression, alter chromatin structure, induce genomic rearrangements, and influence trait formation [8,9,10]. Research suggests that TEs might mediate responses to biotic and abiotic stress by acting as metastable epialleles [7,11,12]. Recent advancements in long-read sequencing have enhanced genome assemblies, improving our understanding of TEs’ impact on genome structure and gene regulation.

TEs are categorized into two classes based on their transposition mechanisms: retrotransposons and DNA transposons. Retrotransposons, which include endogenous retroviruses (ERVs), long interspersed nuclear elements (LINEs), and short interspersed nuclear elements (SINEs), are particularly abundant in mammalian genomes, using a copy-and-paste mechanism [13]. LINEs, transcribed by RNA polymerase II, copy themselves into mRNA-like transcripts and integrate into the genome with the aid of proteins encoded by ORF1 and ORF2 [14]. SINEs, which do not encode proteins for retrotransposition, rely on the reverse transcriptase and endonuclease from other TEs, such as LINEs [15]. A recent study assessed TE diversity across 248 placental mammal genomes, revealing that LINEs constitute 8.2–52.8% and SINEs 0.4–32.1% of the genome [16]. The dominant LINE family in mice and humans is LINE-1, comprising 19% and 17% of their genomes, respectively [17]. The most abundant SINE family in humans is Alu repeats, accounting for approximately 11% of the human genome, while B1 elements dominate in mice, constituting around 2.7% of the mouse genome [18,19]. Most LINEs and SINEs have lost their ability to produce a functional protein that makes them unable to retrotranspose [20]. As research on TEs progresses, evidence increasingly shows that LINEs and SINEs directly or indirectly affect the genome through mechanisms such as modified gene regulatory networks and induced genomic rearrangements [12,21].

LINEs and SINEs drive eukaryotic genome evolution. LINEs, particularly LINE-1 sequences, evolve as dominant lineages responsible for retrotransposition, with new master elements regularly replacing older ones [22]. In humans, the LINE-1 family has amplified significantly since the ancestral human and mouse lineages diverged approximately 65–75 million years ago [23]. SINEs like B1s, specific to rodents and interacting with LINEs for over 100 million years, exhibit remnants of extinct families in mammalian genomes, providing insights into retrotransposon evolution [24]. The evolution of LINEs and SINEs is influenced by their emergence, mobilization mechanisms, and population genetic processes such as natural selection and genetic drift [15,25]. For instance, Kido et al. reported a surge in retrotransposon dispersion associated with the formation of salmonid species [26]. Evolutionary analyses of TEs have revealed that the activation of LINE and SINE families induces intraspecific polymorphisms and affects adaptation, population stratification, selection footprints, and diversity within and among species [27,28,29].

Recent findings indicate that homotypic interactions between TEs of the same family may regulate higher-order genome structures [12,30,31]. SINEs and LINEs are preferentially enriched in euchromatic A compartments and heterochromatic B compartments, respectively. Román et al. reported that the B1 SINE retrotransposon B1-X35s possesses strong intrinsic insulator activity [32], regulated by binding to transcription factors such as AHR and SLUG (SNAI2). This insulator activity is associated with the barrier protein CTCF, which is enriched at the boundaries of topologically associating domains (TADs) and chromatin loops [33,34]. TEs locally generate novel anchoring motifs that facilitate species-specific loop formation and stabilize conserved loops across species through a novel mechanism for CTCF binding site turnover [35]. A follow-up study confirmed these findings and further discovered that SINE sequences overlap with approximately half of CTCF-binding sites in mice and one-third in humans [9]. Choudhary et al. (2023) revealed that TE-derived 3D chromosomal structures (e.g., loop anchors and TAD boundaries) are lineage-specific across mammalian species [35]. Notably, the abundance of LINEs and the number of LINE-derived CTCF sites diminish at TAD boundaries due to LINE length limitations [36].

In addition to regulating gene expression through chromatin interactions, transposable elements (TEs) contribute to gene regulatory networks and induce epigenetic modifications by influencing gene transcription, pre-mRNA splicing, or mRNA stability [12]. One classic example is LINE-1, which represses gene transcription by sequestering genes into a silent nuclear compartment, a process it participates in during X chromosome inactivation [37]. TEs can directly regulate gene transcription by generating various types of alternative splicing, such as intron retention, exon skipping, and exonization, leading to novel mRNA isoforms or premature termination codons [38,39]. Another significant mechanism through which TEs influence gene expression is by disrupting cis-regulatory elements, such as promoters, enhancers, silencers, and insulators [40,41]. For instance, SINE insertions within gene introns may provoke ectopic splicing by introducing alternative splicing sites or inducing premature transcript termination by interfering with RNA polymerase II and promoter interactions [42,43]. Increasing evidence also shows that many noncoding RNAs (ncRNAs) are derived from TEs [44,45].

In this study, we generated a comprehensive, high-resolution map of transposable elements (TEs) in hedgehogs, specifically classifying LINE and SINE families using a polygenic consensus tree. We analyzed the genomic distribution of LINEs and SINEs within the *A. albiventris* genome, revealing their prevalence in intergenic regions, gene-rich regions, and within genes, suggesting potential regulatory influences. Using Hi-C (High-throughput Chromosome Conformation Capture) and HiFi (High-Fidelity Long-Read Sequencing), we systematically examined the 3D chromatin architecture, GC content, and DNA methylation patterns associated with these TE families, addressing how they might shape genomic organization. Furthermore, through RNA-seq mapping, we quantified TE expression levels and identified candidate genes potentially associated with expressed TEs, raising the possibility that TE activity could influence spine formation. Our findings provide new insights into how TEs contribute to 3D genome structure and gene regulation in *A. albiventris* evolution.

## 2. Methods

### 2.1. Extraction of LINEs and SINEs

The amount and reference composition of repetitive elements were investigated according to the following protocol:

(i) Reference database: RepeatModeler v2.0.215 was used to perform ab initio repeat family prediction from the *A. albiventris* genome [46]. Unknown classification of the ab initio prediction was performed using TEclass v2.1.3d [47]. The predicted repeats from ab initio, Dfam database, and the Repbase library were merged and clustered using cd-hit-est (parameters: -n 5, -aL 0.99, -c 0.8 and -s 0.8) to create a new repetitive sequence library [48].
(ii) (TE annotation: Repeats in the *A. albiventris* were detected and classified by searches for similarity to sequences in the reference repeat database with RepeatMasker v4.0.7 (http://www.repeatmasker.org, accessed on 11 September 2024) using default settings. We extracted LINE and SINE annotation information from the RepeatMasker output by filtering based on the classification qualifier.
(iii) TE consensus sequence: To obtain the consensus sequence for LINE and SINE families, we followed the pipeline proposed by Goubert et al. (2022) [49]. First, each raw sequence of LINE and SINE from the output was mapped onto the genome using BLAST v2.15 [50], applying a 95% similarity threshold, which has been commonly used in repeat annotation pipelines to ensure that only closely related copies are considered. Next, the putative copies of the query sequence were used as input for the multiple alignments using mafft v7.520 software with default parameters [51]. Gaps, rare insertions, and highly divergent sequences were removed using the t-coffee tool [52]. Finally, the cons function from the EMBOSS package (http://emboss.open-bio.org/rel/dev/apps/cons.html, accessed on 15 July 2024) was used to generate a consensus sequence that serves as a representative model for each subfamily, not a specific genomic locus.

### 2.2. Genome Feature of LINEs and SINEs

The Manhattan plot of LINEs and SINEs density (number and length) within 100 kb windows along chromosomes were visualized using an R script (https://github.com/SystemBio-Sdut/Ata_TEs/, accessed on 21 March 2025). To explore the clustering trend of LINEs and SINEs across the genome, we counted the number and length of neighboring elements per type in 500 kb non-overlapping windows. The LINE and SINE GFF annotation files were used as input data for agat software v1.4.2 to convert gene coordinates in BED format [53]. The annotatePeak and upsetplot function in ChIPseeker package v.1.18.0 was used to determine and visualize the genomic context (Intergenic, Promoter, Intron, Downstream, 5UTR, CDS, 30UTR) of the LINEs and SINEs [54], respectively. Based on the positions of the TEs and genes, we calculated the distance between each TE and its nearest genes. Genes adjacent (within 5 kb, both upstream and downstream) to each element of LINE and SINE are defined as LINE-enriched and SINE-enriched genes. The raw LINE and SINE element matrices containing repeat percentages in genic regions for each gene were calculated according to the methods described by Lu et al. (2020) [30]. Subsequently, the matrix was normalized using the quantile method implemented in the R package pre-processCore [55]. The hierarchical clustering was performed in R using the normalized matrix using the hclust function with the average method, and clusters were visualized using R scripts. To investigate whether LINEs and SINEs could affect gene function, the GO enrichment result of LINE-enriched and SINE-enriched genes was compared to that of random gene sets, and the corresponding cumulative distribution curve (CDC) of *p*-values was plotted. Differences in CDC between enriched and randomly selected genes were assessed using the Wilcoxon test. The Wilcoxon test was selected as it is a non-parametric test that does not assume a specific data distribution. This makes it suitable for comparing TE enrichment levels across different genomic regions, allowing us to determine whether certain genomic features are significantly associated with TE accumulation.

### 2.3. Insert Age Estimation

To estimate the age of LINE and SINE insertions in *A. albiventris*, we performed a copy-divergence analysis of the LINE and SINE consensus sequences using the Kimura 2-parameter distance model. Kimura distances between genome copies and consensus sequences from the repetitive library were calculated using buildSummary.pl, calcDivergenceFromAlign.pl, and createRepeatLandscape.pl script based on alignment files generated by RepeatMasker v4.0.7. Activity periods were estimated using an average mammalian genome mutation rate of 2.2 × 10^−9^ substitutions per site per year [56]. Distribution histograms for sequence divergence of LINE and SINE were drawn using a bin size of 5. Putatively active copies were defined based on younger insertion ages (<15 Mya) and higher copy numbers.

### 2.4. Phylogenetic Tree and Family Classification for LINEs and SINEs

LINE and SINE consensus sequences were used to construct phylogenetic trees, respectively. Multiple sequence alignment of the consensus sequences was generated using mafft v7.520 [51], and the resulting alignment file was used to create a maximum-likelihood tree with FastTree v2.1.11 [57]. The phylogenetic tree was visualized with the ggtree v3.10.1 [58]. Family classification was performed using the COSEG program (http://www.repeatmasker.org/COSEGDownload.html, accessed on 11 September 2024). Each family was assigned a unique identifier using the format “family-X_yYyy”, where X is a unique number, and yYyy is a four-letter identifier for the species.

### 2.5. Hi-C Analysis and 3D Chromatin Architecture

The Hi-C data for *A. albiventris* was previously used in genome scaffolding, and the summary statistics for its quality control were described [3]. Paired-end raw reads of Hi-C library data were processed using the process recommended by Omin-C^TM^. The Hi-C contact matrices were generated using the hicBuildMatrix tool in HiCExplorer v3.7.2 with different bin sizes and then normalized and KR balanced afterward using hicNormalize and hicCorrectMatrix [59], respectively. A/B compartments were called using the hicPCA tool on 100 kb binned matrices based on the first principal component (PC) of the Pearson correlation matrices of each chromosome generated from the Hi-C map. The TADs and boundaries were identified using the hicFindTADs tool with the default parameters “—minDepth 15,000—maxDepth 75,000—step 7500—delta 0.01—thresholdComparisons 0.01—correctForMultipleTesting fdr”. We identified chromatin loops at 100 kb resolution.

### 2.6. DNA Methylation

DNA methylation data were obtained from HiFi sequencing reads, and quality control was performed using pbccs to generate high-fidelity reads. These details have been incorporated into the Materials and Methods section to ensure transparency and reproducibility. The whole genome 5mCpG methylation was identified using a deep learning method called ccsmeth v0.3.2 [60], based on kinetics features from PacBio HiFi reads, which were generated from muscle tissue. First, the *ccsmeth align_hifi* command was used to align the HiFi reads to the reference genome. Next, methylation predictions were generated using the ccsmeth call_mods command with model ‘model_ccsmeth_5mCpG_call_mods_attbigru2s_b21.v1.ckpt’. Methylation frequency was obtained at the genome level by using ccsmeth call_freqb with the modbam files.

### 2.7. Transcriptomic Analysis of LINEs and SINEs

To further explore the role of LINEs and SINEs on gene regulation, we analyzed a published transcriptome dataset involved in spine development in *A. albiventris* to evaluate the genome-wide expression levels of LINEs, SINEs, and genes. This dataset, originally published by Li et al. (2019) [48], assessed genome-wide gene expression in dorsal and abdominal skin tissues across three developmental stages: Stage I (within 2 h of birth), Stage II (after 2 h but within the first day of birth), and Stage III (5 days after birth), based on the de novo assembly of the *A. albiventris* transcriptome. These data were generated using a traditional RNA-seq paired-end sequencing approach with three biological replicates, and the library was constructed using a standard poly(A)-enriched RNA-seq method. RNA-seq data quality was assessed using fastp for read quality checks (ref). We employed a modified version of REdiscoverTE, originally developed for genome-wide TE expression analysis in human RNA sequencing data [61], to quantify the expression levels of LINEs, SINEs, and genes in the *A. albiventris* transcriptome. First, we classified LINEs and SINEs into three categories according to their genomic locations using the *annotatePeak* function from the ChIPseeker package [54]: intronic, exonic, and intergenic elements, with intergenic regions defined as being at least 5000 bp from any gene. Next, Salmon v1.2.0 was used to generate the quasi-mapping index for the REdiscoverTE reference transcriptome of *A. albiventris* and to quantify RNA-seq data [62], with parameters set to account for GC content bias and sequence-specific bias. Third, the LINEs and SINEs within the intergenic regions, introns, and exonic regions, along with the gene expression levels, were calculated using a *rollup* R script in the REdiscoverTE program. The transcript-per-kilobase-million (TPM) quantification results generated by this script were then used for subsequent downstream analyses. Furthermore, high-confidence expressed genes, LINEs, and SINEs were identified using the *filterByExpr* function from the R package edgeR v4.0.16 [63]. Differentially expressed genes (DEGs), differentially expressed LINEs (DELs), and differentially expressed SINEs (DESs) between the time-series gene expression profiles of abdomen hair and dorsal spine tissues were identified using the maSigPro v1.70 of the Bioconductor software with adjusted *p*-values of ≤0.05 and *R*-squared value of ≥0.6 [64].

### 2.8. Statistical Analysis and Data Visualization

All the statistical analyses and visualization were implemented in the R v4.2.2 statistical software (http://www.R-project.org, accessed on 15 October 2023). The randomness of LINE and SINE distribution in the genome was tested using a Wald–Wolfowitz runs test at a 5% significance [65]. The Wald–Wolfowitz runs test was chosen because it is a non-parametric method used to assess the randomness of data distribution. GO enrichment analysis was performed using clusterProfiler v4.6.2 package with an adjusted *p*-value cut-off of 0.01 [66]. The analysis employed the hypergeometric test to identify significantly overrepresented GO terms. Pearson correlation analysis was performed using *cor.test* function. Student’s *t*-test was performed using the *t*-test function. Histograms, barplot, boxplots, and heatmaps were plotted using the built-in function of R software. Venn diagrams were drawn using the package venn v1.11.

## 3. Results

### 3.1. Distribution Feature of LINE and SINE Elements in the A. albiventris Genome

**Figure A1:**
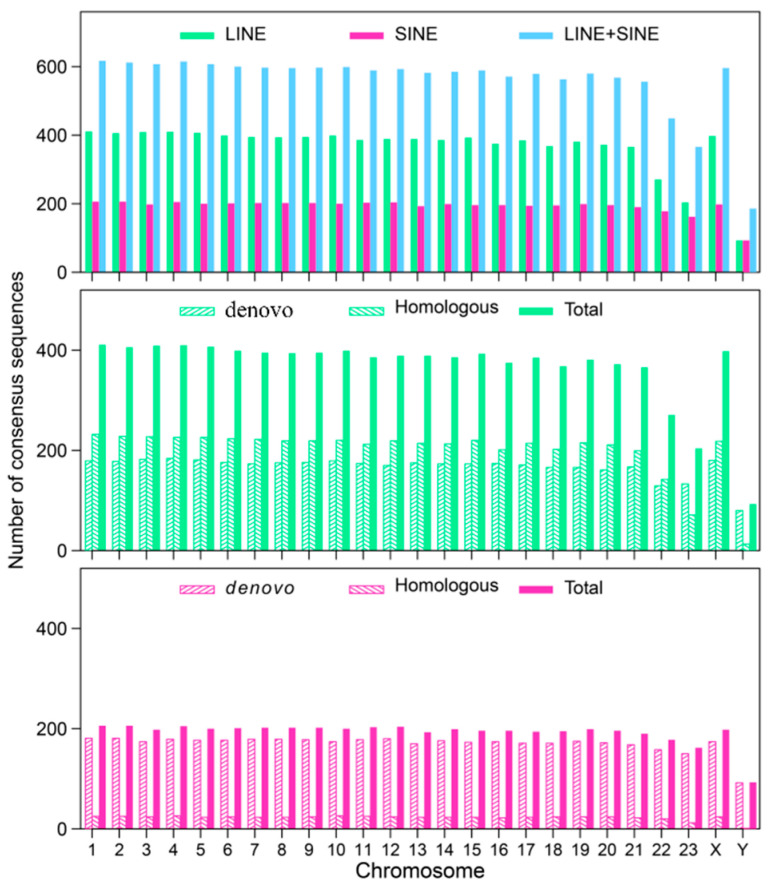
The summary of LINE and SINE annotations in the *A. albiventris* genome includes three plots: the top plot displays the number of consensus sequences for LINEs and SINEs; the middle plot shows the number of de novo and homologous alignments for LINEs; the bottom plot presents the number of de novo and homologous alignments for SINEs.

**Figure A2:**
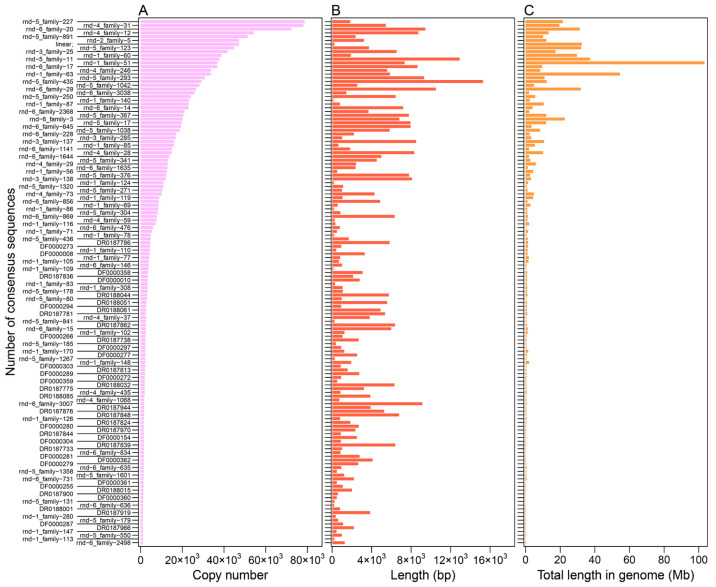
The copy number (**A**), length (**B**), and proportion of genome (**C**) of the LINE consensus sequences.

**Figure A3:**
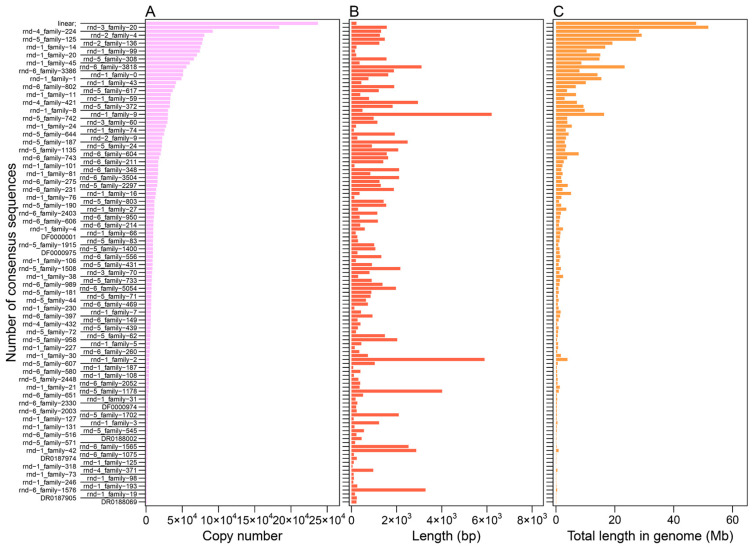
The copy number (**A**), length (**B**), and proportion of genome (**C**) of the SINE consensus sequences.

To systematically investigate the components and distribution of LINEs and SINEs in the *A. albiventris* genome, we employed the RepeatMasker tool, integrating data from RepBase and de novo databases. Our findings revealed that the number of consensus sequences identified through homologous annotation for LINEs was slightly higher than those based on de novo predictions in most chromosomes. In contrast, for SINEs, the number of sequences identified by de novo prediction was significantly greater than those identified through homologous searching (Figure A1). The analysis of consensus sequences from specific LINE and SINE subfamilies revealed that an increase in the copy number of these sequences significantly contributes to genome expansion (Figure A2 and Figure A3). These sequences, which belong to distinct subfamilies, may vary in their evolutionary age and activity, influencing their role in shaping the genome. For example, the top two SINE consensus sequences with the copy number are ‘linear’ and ‘rnd-4_family-31’, covering 1.71% (~47.62 Mb) and 1.86% (~51.75 Mb) of the genome, respectively. Further sequence alignment results indicated that while LINEs contribute a larger percentage to the genome due to their longer sequence lengths, SINEs are more abundant in terms of copy number. Specifically, LINEs, primarily from the LINE1 family, account for 27.35% (~724.78 Mb) of the *A. albiventris* genome, with a total of 1,656,651 copies. In contrast, SINEs, mainly comprising the tRNA family, represent 20.45% (~541.99 Mb) of the genome, with approximately 2.5 million copies, making them the most abundant transposable elements in this genome (Table 1). The genomic content of LINEs in *A. albiventris* is generally similar to that of the majority of mammals, whereas the SINE content is substantially higher than that of other mammals.

**Figure 1:**
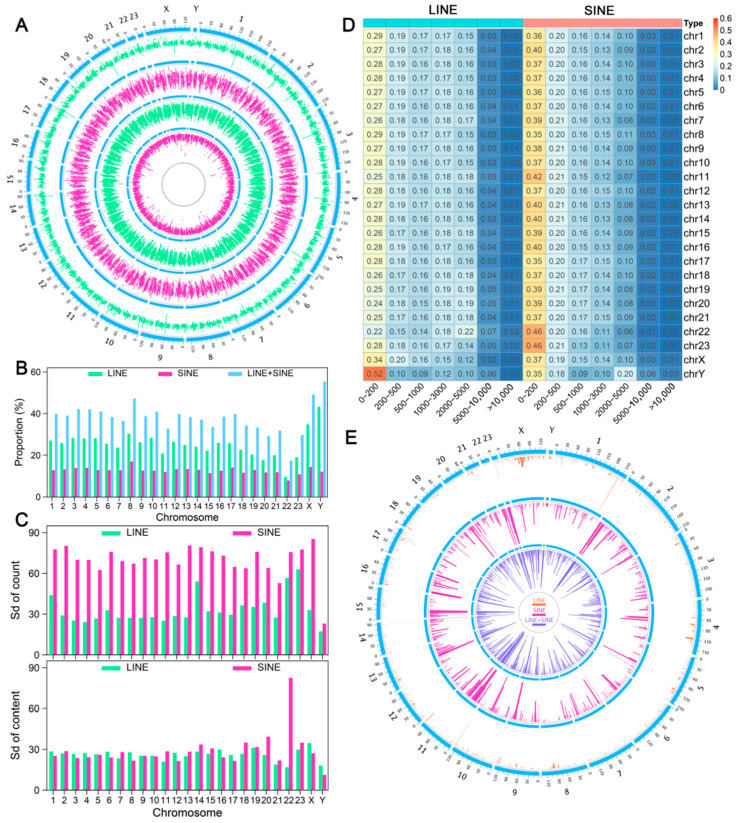
Distribution characteristics of LINEs and SINEs in the *A. albiventris* genome. (**A**) Distribution of LINE and SINE counts and sequence lengths across 250 kb genomic windows, arranged (outer to inner): LINE count, SINE count, LINE length, and SINE length. Green indicates the distribution of LINEs (long interspersed elements); pink indicates the distribution of SINEs (short interspersed elements); blue is used to mark chromosome locations. (**B**) Histogram displaying the proportions of LINEs and SINEs across each chromosome. Green bars indicate LINE proportions, pink bars represent SINE proportions, and blue bars show the combined proportion of LINEs and SINEs. (**C**) Standard deviation (SD) of LINE and SINE sequence lengths and count across chromosomes. The top panel shows SD for counts, while the bottom panel shows SD for sequence lengths. Green and pink bars correspond to LINEs and SINEs, respectively. (**D**) Proportion of LINEs and SINEs at varying proximity distances. Rows represent chromosomes, and columns indicate distance categories. Warmer colors denote higher proportions. (**E**) The genomic distribution of LINE and SINE blocks. LINE blocks are marked in blue, and SINE blocks are marked in pink. Genomic coordinates are arranged circularly around the plot.

**Figure A4:**
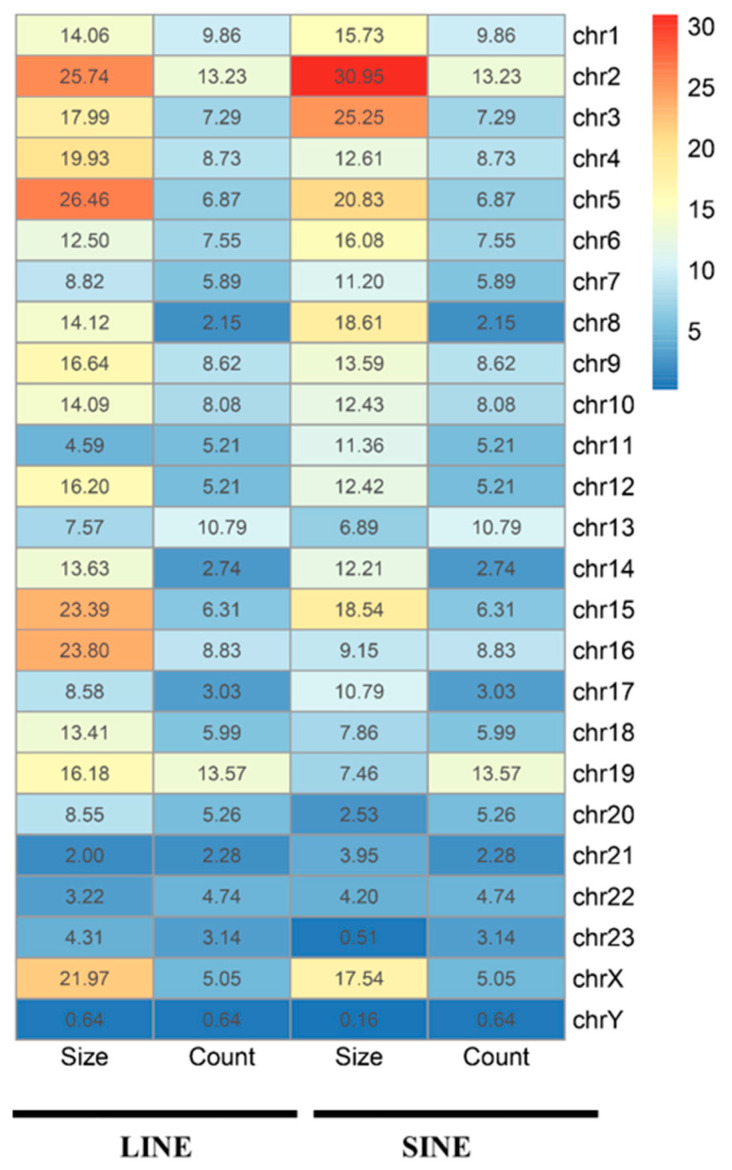
The heatmap shows the logarithms of *p*-values from random distribution tests of LINEs and SINEs.

Using 250 kb bins, we analyzed the total sequence length and count of LINEs and SINEs within each bin across the *A. albiventris* genome to observe their distribution patterns. LINEs exhibited a non-random distribution across most chromosomes (*p* < 0.01), except for the Y chromosome, where the distribution was random (Figure 1A and Figure A4). SINEs showed a similar pattern to LINEs, except for autosome 23, where the sequence length per bin differed. Notably, LINEs displayed large fluctuations in sequence length per bin on some chromosomes, especially the sex chromosomes, while SINEs showed relatively stable values in both sequence length and count per bin across all chromosomes (Figure 1B).

The One-Sample Student’s *t*-test results indicated that the ratio of LINE or SINE sequences in each chromosome followed a uniform distribution (*p* > 0.05). We further calculated the standard deviation (SD) of the sequence length and count of LINEs and SINEs across all 250 kb bins within each chromosome. The SD of sequence length was similar between chromosomes and between LINEs and SINEs, except for SINE on autosome 22. However, there was significant variation in SD values among chromosomes for the number of LINEs and SINEs, with the SD values for LINE count being much greater than for SINE count across the entire genome (Figure 1C). These findings indicate that the distribution of SINEs differs significantly from that of LINEs in the *A. albiventris* genome.

To explore this further, we analyzed the distance between consecutive TEs of the same type along the chromosome. We found that the proportion of SINEs with proximity distances between 0 and 200 bp ranged from 35% to 40% across different autosomes, significantly higher than the proportion of LINEs (24–29%) (Figure 1D). To analyze the distribution patterns of SINEs and LINEs, we created a 500 kb sliding window for each chromosome. A group of TEs of the same type was defined as a block within the window if the distance between any two consecutive TEs was less than or equal to 1 kb and the number of consecutive TEs exceeded four. SINEs formed significantly more blocks across the genome than LINEs (Figure 1E).

### 3.2. Impact of LINEs and SINEs on Gene Function in A. albiventris

**Figure 2:**
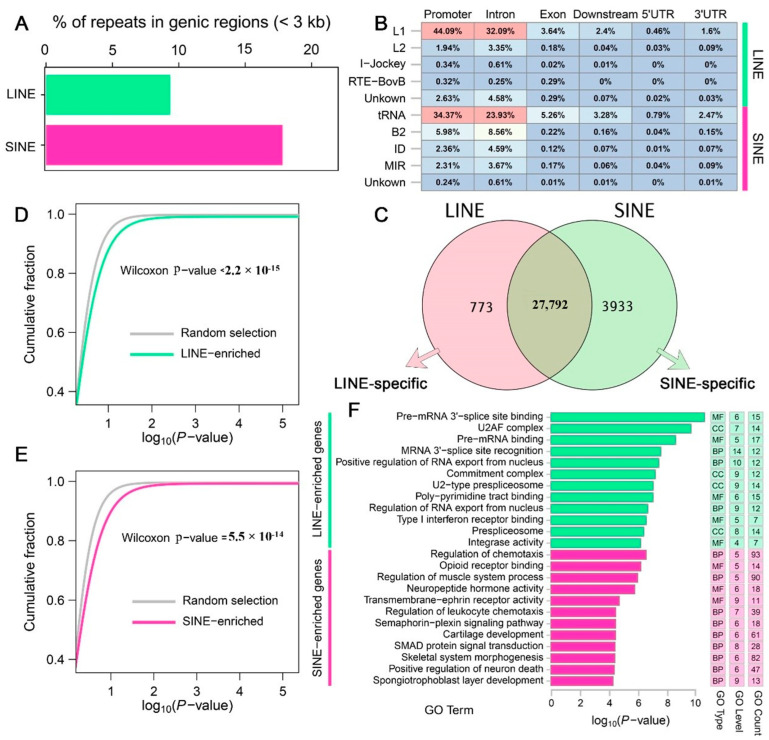
Genic LINEs and SINEs associated with gene function in the *A. albiventris* genome. (**A**) Bar plot showing the proportion (%) of LINEs (green) and SINEs (pink) located near genic regions (within 3 kb). (**B**) Genomic annotation of LINE and SINE families within genic regions. The heatmap displays the proportions of these elements in specific genomic features, including promoters, introns, exons, downstream regions, 5′ UTRs, and 3′ UTRs. Color intensity reflects the relative abundance of each repeat type in the corresponding genomic feature. Different colors indicate the relative abundance of transposon (TE) types: red represents the highest proportion, yellow/white represents a medium proportion, and blue represents a low proportion. The green and pink bars on the right mark the LINE and SINE categories, respectively, for easy classification visualization. (**C**) Venn diagram illustrating the overlap between gene sets associated with LINEs (pink) and SINEs (green). The central overlap represents genes enriched for both LINE and SINE elements, while the non-overlapping sections represent LINE-specific and SINE-specific genes. (**D**,**E**) Cumulative distribution curves (CDC) comparing GO analysis of genes neighbored by LINEs and SINEs versus random gene sets. (**F**) GO enrichment analysis of LINE-specific and SINE-specific genes defined in (**C**).

**Figure A5:**
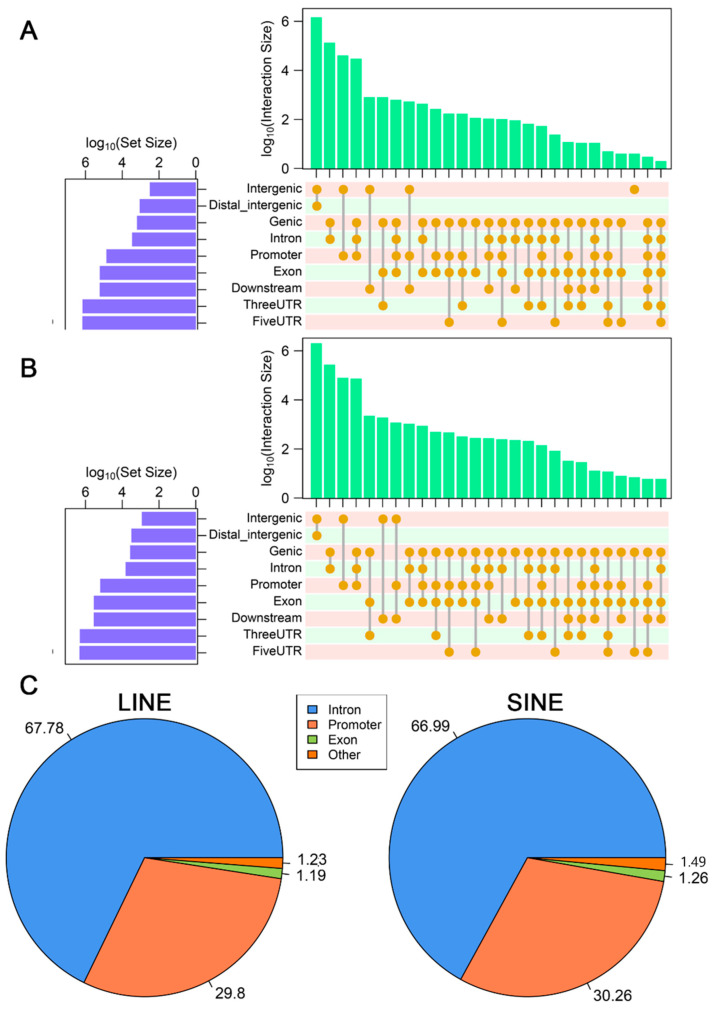
The genomic annotation of LINEs and SINEs: (**A**) Upset plot of annotation information of LINEs. (**B**) Upset plot of annotation information of SINEs. (**C**) Pie plot of annotation information of LINEs and SINEs. Some categories may not be included in the pie chart, causing the total to be less than 100%.

To investigate the relationship between the composition and distribution of LINE and SINE retrotransposons in the *A. albiventris* genome and gene function, we analyzed the intersection between their chromosomal localization and genic regions (±3 kb of a gene). We found that 9.36% (155,110) of LINEs and 17.82% (440,482) of SINEs overlapped with protein-coding genes (Figure 2A). The genomic annotation of LINE and SINE sequences in genic regions revealed they are highly enriched in regulatory regions, such as promoter and intron (Figure A5A,B). Specifically, 29.79% of LINEs and 30.26% of SINEs were located in promoter regions, while 67.78% of LINEs and 66.99% of SINEs were located in intron regions (Figure A5C). A heatmap depicting the proportion of LINEs and SINEs families covering different genic features indicated that over 80% of L1 and 5.3% of L2 families were concentrated in regulatory regions located within ±3 kb of a gene, while 64.84% of tRNA and 15.94% of B2 are present within this range for SINEs (Figure 2B). Additionally, 3.64% of L1 and 5.26% of tRNA families were enriched in exons of mRNAs. These results suggest that genic retrotransposons of the *A. albiventris* genome are mainly clustered in promoter and intron regions.

Retrotransposons displayed extensive impact on the genic regions. We observed that 79.91% (28,565) of genes overlapped with LINEs, and 88.75% (31,725) of genes overlapped with SINEs out of a total of 35,746 genes (Figure 2C). Approximately 773 and 3933 genes were identified as LINE-specific or SINE-specific genes, respectively. These specific genes are more likely to be enriched in particular biological functions compared to randomly selected equal numbers of genes (Figure 2D). For example, LINE-specific genes are significantly enriched in RNA-related “alternative splicing” functions, including Pre-mRNA 3′-splice site binding, U2AF complex, Pre-mRNA binding, and regulation of RNA export from the nucleus, which are crucial for mRNA processing. In contrast, SINE-specific genes were strongly enriched in specialized functions such as regulation of chemotaxis, regulation of muscle system process, cartilage development, and skeletal system morphogenesis (Figure 2E).

### 3.3. Evolution of LINEs and SINEs in the A. albiventris Genome

**Figure A6:**
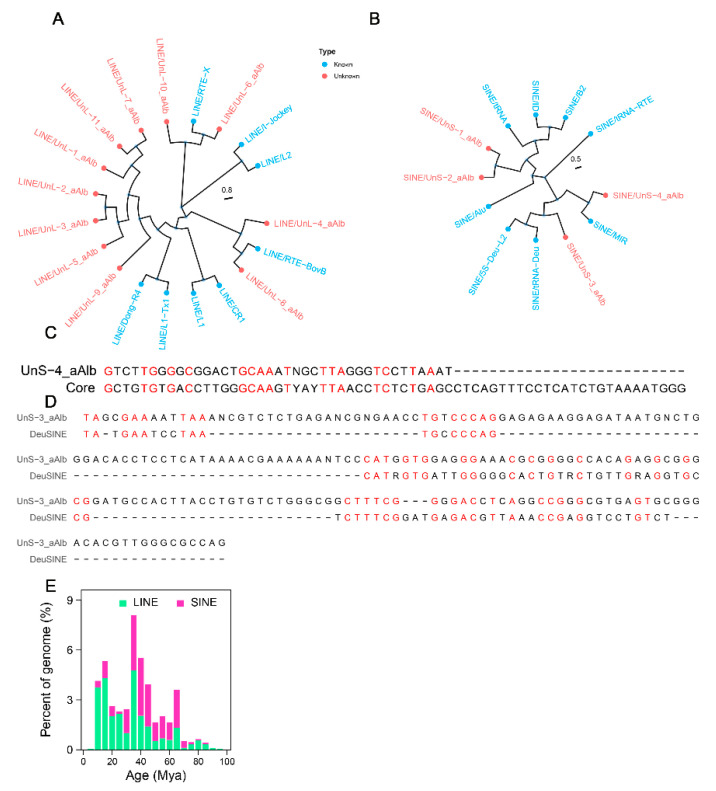
Evolution and activity analysis of LINEs and SINEs in the *A. albiventris*: (**A**,**B**) Maximum likelihood phylogenetic tree of both known and novel of LINE and SINE families. (**C**) Similarity between the core region of the SINE family and the non-tRNA fragments of UnS-4_aAlb in *A. albiventris*; Red indicates the labeled genomic regions. (**D**) Sequence alignment of the DeuSINE domain with UnS-3_aAlb sequence. Red indicates the labeled genomic regions. (**E**) Insertion age distribution of LINEs and SINEs.

To systematically explore the evolutionary profile of LINEs and SINEs in the *A. albiventris* genome, we constructed maximum likelihood phylogenetic trees for these elements using their consensus sequences. We identified 11 out of 51 unclassified LINE consensus sequences (each with more than 10 copies and a length exceeding 1000 bp) (Table S1) and integrated them with known LINE families (eight known families) to construct a phylogenetic tree (Figure A6A). Seven of these sequences (UnL-9_aAlb, UnL-5_aAlb, UnL-3_aAlb, UnL-2_aAlb, UnL-1_aAlb, UnL-11_aAlb, and UnL-7_aAlb) were identified as potential members of an unknown LINE family specific to *A. albiventris*. Additionally, UnL-4_aAlb and UnL-8_aAlb are likely members of the RTE-BovB family, while UnL-6_aAlb and UnL-10_aAlb may belong to the RTE-X family (Table S1). For SINEs, we generated a phylogenetic tree based on four unclassified consensus sequences (with over 10 copies each and a length exceeding 100 bp) and eight known SINE families. Our analysis showed that UnS-1_aAlb and UnS-2_aAlb clustered together, indicating their likely membership in a new SINE family (Figure A6B and Table A1). UnS-4_aAlb clustered with SINE/MIR, suggesting it may belong to the MIR family. We further investigated whether UnS-4_aAlb contains the core sequence, a defining feature of the core SINE family [67], and the alignment revealed potential similarity, with notable conservation in the core regions (Figure A6C). Meanwhile, UnS-3_aAlb, which shows some partial similarity to the Due domain (Figure A6D) [68], may represent a SINE family specific to *A. albiventris*. The insertion age analysis revealed that LINEs and SINEs have evolved over a long period, ranging from 5 to 95 million years ago (Mya) in the *A. albiventris* genome, with distinct burst periods and frequencies for each type of TEs (Figure A6E). SINEs were the earliest to expand in the genome, with their first expansion occurring around 65 Mya, contributing to 4% of the genome. However, the majority of SINEs originated from amplification events between 35 and 45 Mya. In contrast, LINEs have a more recent evolutionary history, with major activity occurring between 15 and 35 Mya.

**Figure 3:**
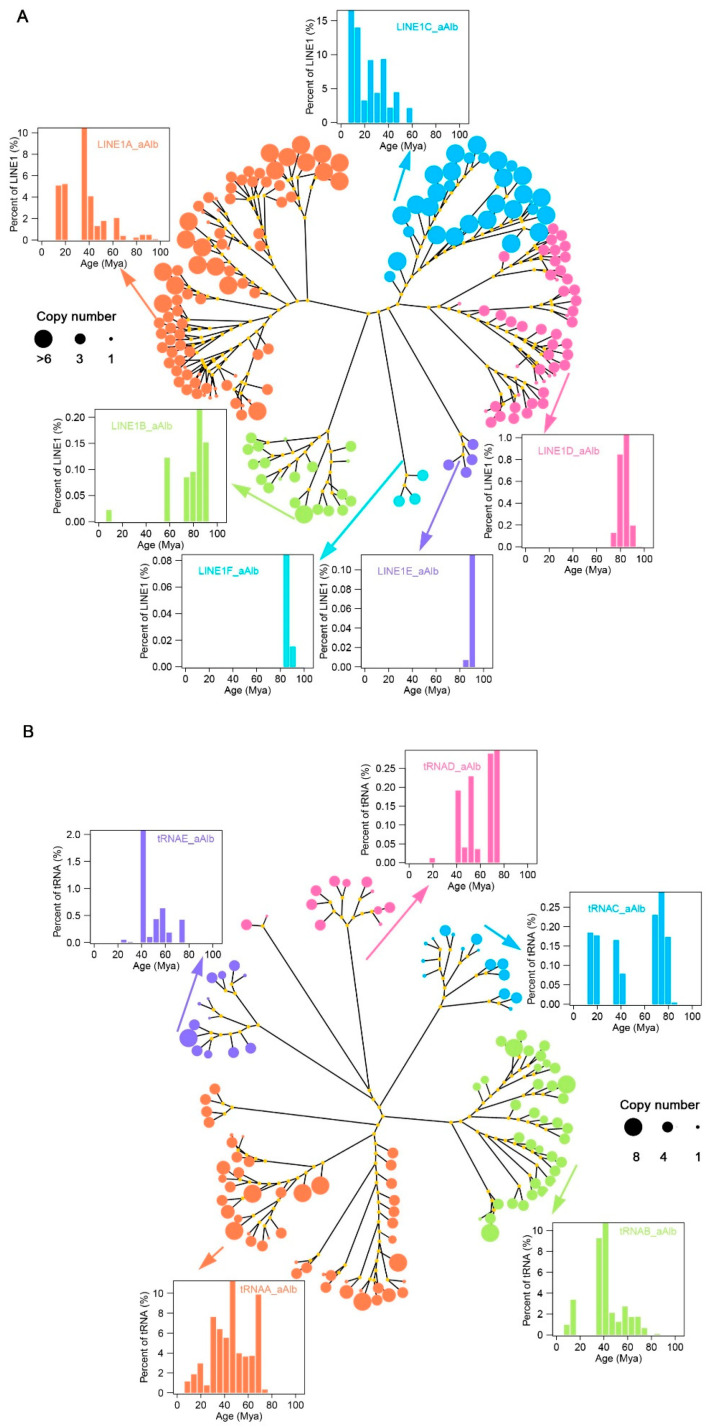
Evolution and activity analysis of LINE1 (**A**) and tRNA-SINE (**B**) subfamilies in the *A. albiventris*. The tree represents the evolutionary relationships among different subfamilies, with branch colors showing individual subfamilies. Node sizes indicate the copy number of each subfamily, with larger nodes representing higher copy numbers. Insets show histograms of the age distribution (millions of years ago, Mya) for specific subfamilies, illustrating their historical activity levels.

**Figure A7:**
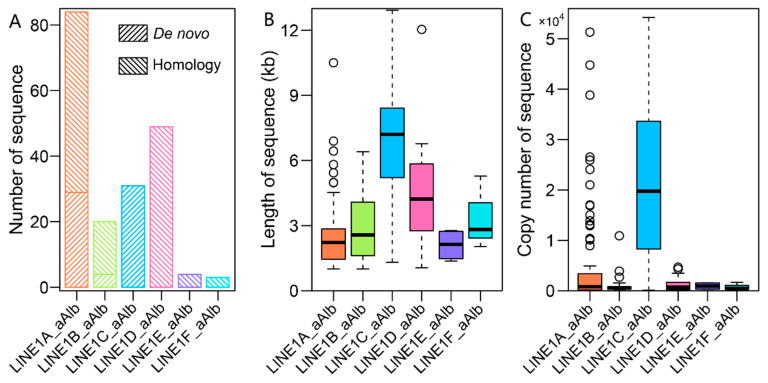
Distribution of the number (**A**), length (**B**), and copy number (**C**) of consensus sequences of different LINE1 subfamilies.

Out of 445 LINE consensus sequences, 191 were classified as high-copy sequences (more than 10 copies per sequence) and were classified under the LINE1 family, which constitutes 86.71% of the total LINE sequence length. These high-copy subfamilies include 64 new consensus sequences generated from the de novo database by RepeatModeler2 software and 127 known subfamilies from the RepBase database (Figure A7A). The 191 consensus sequences were further classified into six subfamilies (termed LINE1A_aAlb, LINE1B_aAlb, LINE1C_aAlb, LINE1D_aAlb, LINE1E_aAlb and LINE1F_aAlb) based on the polygenic consensus tree (Figure 3A). There were significant differences in sequence length, copy numbers, insertion times, and the number of potentially active LINE1 elements among the subfamilies (Figure 3A and Figure A7B,C). The LINE1A subfamily exhibited the highest diversity and longest expansion, with 84 consensus sequences (55 known and 29 unknown), and it also displayed high activity, with many putatively active copies in the genome. The LINE1B, LINE1C, and LINE1D subfamilies contain 20, 31, and 49 consensus sequences, respectively. Most of the putatively active LINE1 elements were found in the LINE1C subfamily, indicating recent activity between 5 and 15 Mya and a high number of active copies. In contrast, ancient subfamilies like LINE1E and LINE1F contained inactive elements, with very few putatively active copies detected.

**Figure A8:**
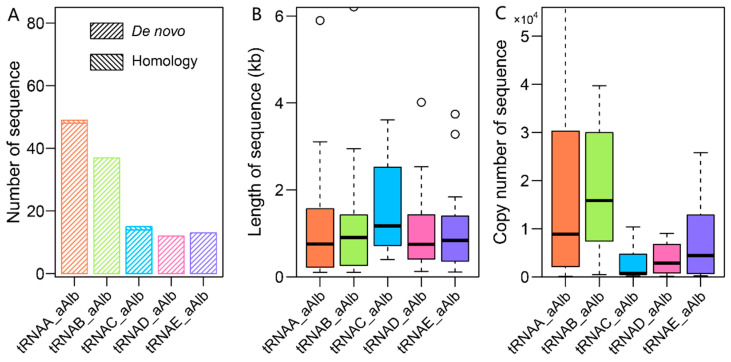
Distribution of the number (**A**), length (**B**), and copy number (**C**) of consensus sequences of different tRNA-SINE subfamilies.

The tRNA-related SINEs make up a subfamily of SINEs, accounting for 96.45% of the total SINE sequence length. Most of the consensus sequences within this subfamily were derived from de novo predictions (Table 1, Figure A8A). We classified 126 tRNA-related SINE consensus sequences (with more than 10 copies) into five subfamilies, tRNAA-aAlb, tRNAB-aAlb, tRNAC-aAlb, tRNAD-aAlb, and tRNAE-aAlb, based on sequence alignment and phylogenetic tree construction (Figure 3B). The tRNAA-related and tRNAB-related SINE families contained 49 and 37 consensus sequences, respectively, ranging from 105 to 6210 bp in length. These two subfamilies contributed 85.33% of the tRNA-related SINE family sequence length, accounting for 38.89% of the total consensus sequences within the tRNA-related SINE family (Figure A8B,C). The tRNAA family has a long evolutionary history, ranging from 5 to 75 Mya, but its activity has significantly declined over the last 25 Mya. In contrast, the tRNAB family also has a long activation period but experienced a burst around 40 Mya, and its activity has diminished in the last 20–30 Mya (Figure 3B). The tRNAC, tRNAD, and tRNAE families were dominant during the ancient evolution of tRNA-related SINEs in the genome, with low ancient copy numbers and weak recent activity.

### 3.4. GC Content Associated with LINEs and SINEs Insertions

**Figure 4:**
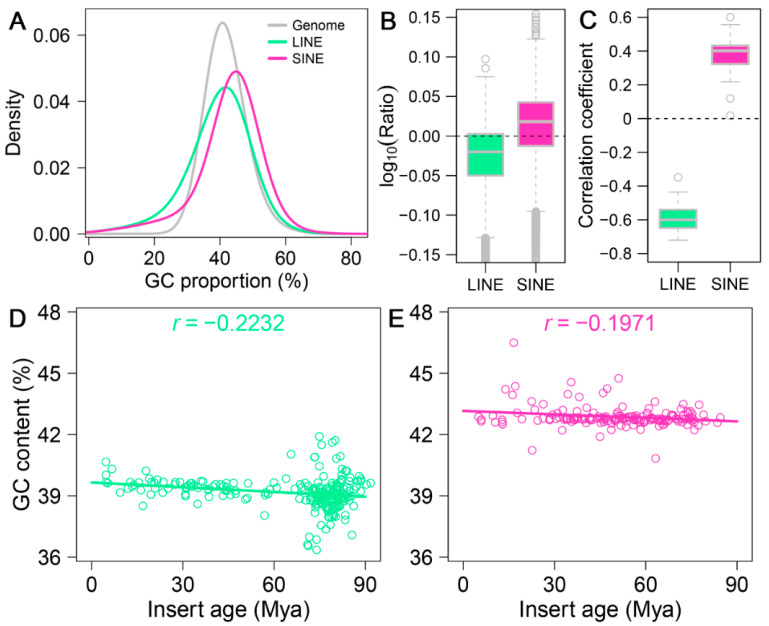
Influence of genomic GC content on retrotransposon distribution in the *A. albiventris* genome. (**A**) Density plot illustrating the GC content distribution for LINEs (green), SINEs (pink), and the entire genome (gray). (**B**) Boxplot representing the log10-transformed GC content ratios of LINEs and SINEs compared to the genome-wide average. The dashed line indicates a ratio of 1 (equal GC content), while deviations highlight enrichment or depletion in GC content. (**C**) Boxplot displaying the correlation coefficients between insert length and GC content for LINEs and SINEs. (**D**,**E**) Scatter plots showing the relationship between retrotransposon GC content and insertion age for LINEs ((**D**), green) and SINEs ((**E**), pink). Each point represents a retrotransposon insertion event, with the fitted regression lines and correlation coefficients (r) indicating the strength and direction of the association.

**Figure A9:**
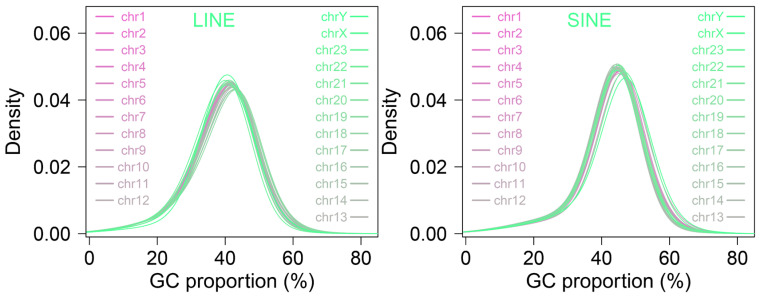
Density plot of GC content of LINEs and SINEs across all chromosomes.

To explore the relationship between GC content and the insertion patterns of LINEs and SINEs, we analyzed the GC content of the elements themselves and their potential effects on these retrotransposons. The comparison of GC content revealed distinct differences in the distribution of GC fractions between LINEs and SINEs, both of which differ significantly from the overall GC ratio distribution across the *A. albiventris* genome (Figure 4A). Specifically, LINEs have a mean GC content of approximately 39%, while SINEs exhibit a slightly higher mean GC content of around 42%. Both LINEs and SINEs demonstrate lighter tails in their GC content distributions, suggesting a lower kurtosis compared to the genomic GC content (Figure A9).

**Figure A10:**
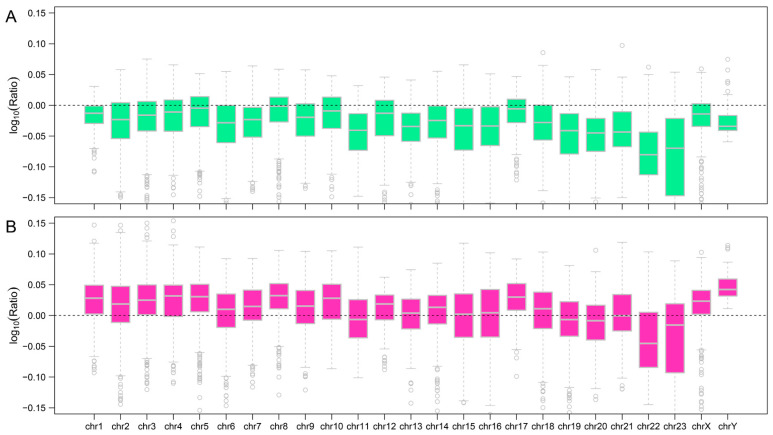
The ratio of LINEs (**A**) and SINEs (**B**) GC content to the whole genome for each chromosome.

**Figure A11:**
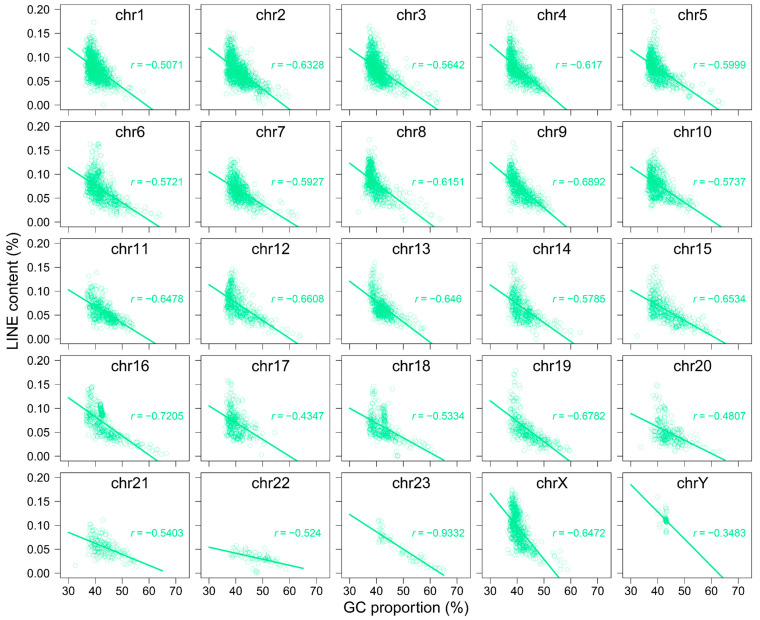
Scatter plots and correlation coefficients between LINE content and its GC proportion for each chromosome.

**Figure A12:**
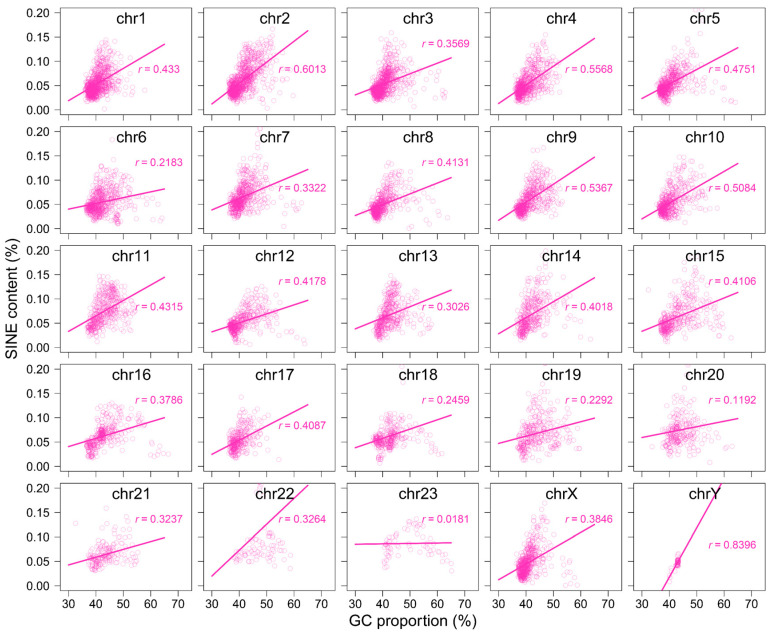
Scatter plots and correlation coefficients between SINE content and its GC proportion for each chromosome.

Using 250 kb sliding windows, we calculated the GC content of retrotransposons themselves within each bin and normalized it by the overall GC content of the corresponding bin. Our results show that LINEs tend to have a lower GC content than the overall genome, whereas SINEs generally display a higher GC content (Figure 4B and Figure A10). Notably, SINEs exhibit lower GC content than the genomic average on specific chromosomes, including 19, 20, 22, and 23 (Figure A10B). To further investigate this pattern, we performed a correlation analysis to explore the relationship between GC content and the insertion lengths of LINEs and SINEs. The analysis revealed a significant negative correlation (r = −0.5996) between genomic GC content and the insertion lengths of LINEs, whereas a significant positive correlation (r = 0.3868) was observed for the insertion lengths of SINEs (Figure 4C, Figure A11 and Figure A12). These findings suggest that LINE insertions contribute to a reduction in GC content within the regions they occupy, while the insertion patterns of SINEs are influenced by GC composition in a different manner than LINEs.

**Figure A13:**
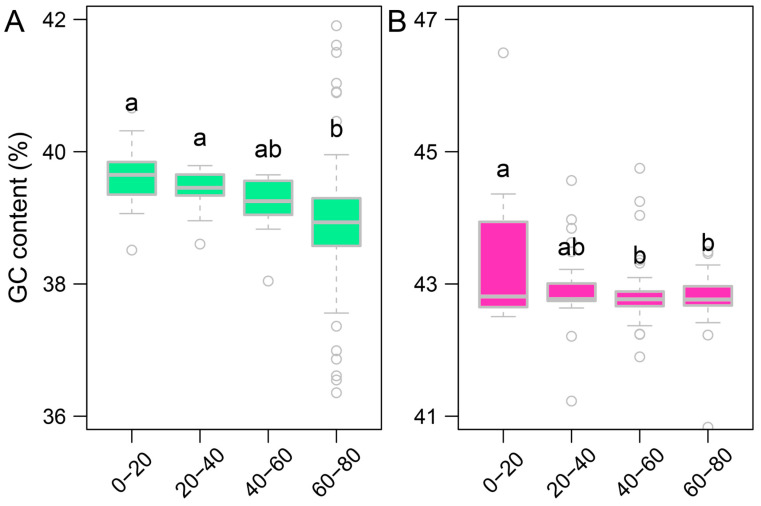
The boxplot of GC content of LINEs (**A**) and SINEs (**B**) in different insert intervals. Lowercase letters in the plot indicate the differences among the insert intervals for GC content.

As shown in Figure 4D,E, we observed weak negative correlations between GC content and insertion age for both LINEs and SINEs, indicating that older LINE and SINE insertions tend to have slightly higher GC content compared to younger insertions. This trend is particularly evident for LINEs, where the higher GC content of older insertions might reflect selective retention in regions with relatively higher GC content rather than a uniform insertion bias. For SINEs, although their GC content generally remains stable across insertion ages, we observed that younger SINEs (0–20 Mya) exhibit significantly higher GC content than older SINEs, as shown in Figure A13. This suggests evolutionary shifts in GC preference over time, highlighting distinct evolutionary dynamics for LINEs and SINEs.

### 3.5. DNA Methylation Patterns of LINEs and SINEs in the A. albiventris Genome

**Figure 5:**
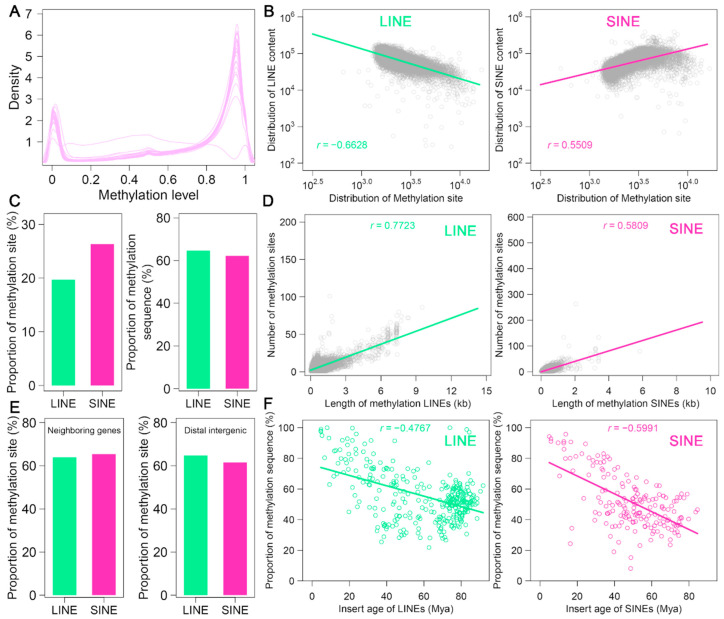
Methylation landscape of LINEs and SINEs in the *A. albiventris* genome: (**A**) The distribution of DNA methylation levels for LINEs (green) and SINEs (pink) across the genome. (**B**) Relationships between LINE (**left**, green) and SINE (**right**, pink) sequence length and the number of methylation sites. Trend lines and correlation coefficients (r) quantify the strength and direction of these associations. (**C**) Bar charts compare the percentage of methylation sites located within LINEs and SINEs (**left**) and the proportion of LINE and SINE sequences that are methylated (**right**), emphasizing contrasts in methylation enrichment. (**D**) The correlation between the length of methylated sequences and the number of methylation sites is shown for LINEs (**left**, green) and SINEs (**right**, pink). (**E**) Proportion of methylation sites near gene regions versus distal intergenic regions for LINEs and SINEs. (**F**) Scatter plots demonstrate the decline in the proportion of methylated sequences with increasing insertion age for LINEs (**left**, green) and SINEs (**right**, pink).

**Figure A14:**
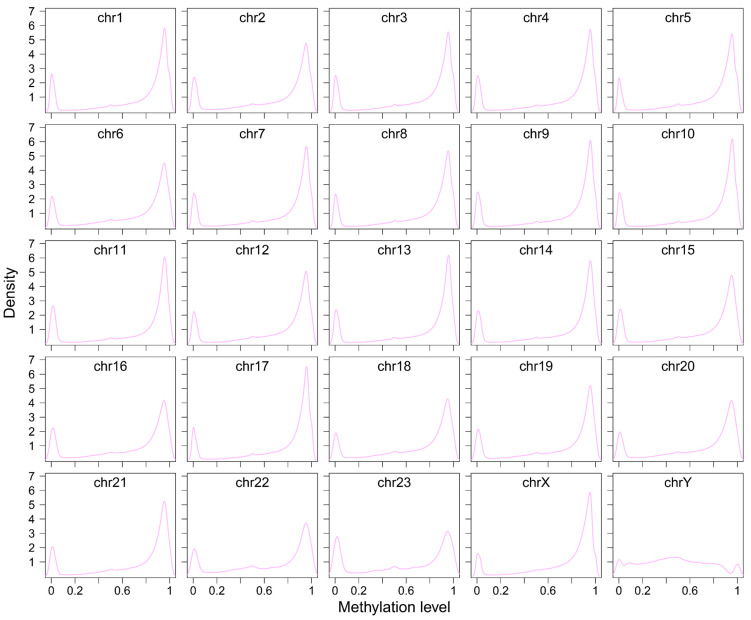
The density plot of methylation levels for each chromosome.

DNA methylation changes within repetitive elements are closely associated with chromatin structure and gene regulation in higher organisms. In this study, we utilized HiFi sequencing data to detect genome-wide 5-methylcytosine (5-mc) methylation in *A. albiventris*. Methylation frequency was calculated as the proportion of methylated reads at each cytosine site, providing a measure of methylation levels. A total of 3,035,188 5-mc methylation sites were identified at single-nucleotide resolution, representing approximately 1.12% of the total genome. Upon analyzing the distribution of 5 mc methylation levels, a bimodal pattern emerged, indicating the presence of two distinct peaks corresponding to low and high methylation levels (Figure 5A and Figure A14). This suggests two different subpopulations of genomic regions with divergent methylation profiles. Notably, the Y chromosome exhibited lower levels of 5 mc methylation compared to the autosomes (Figure A14).

**Figure A15:**
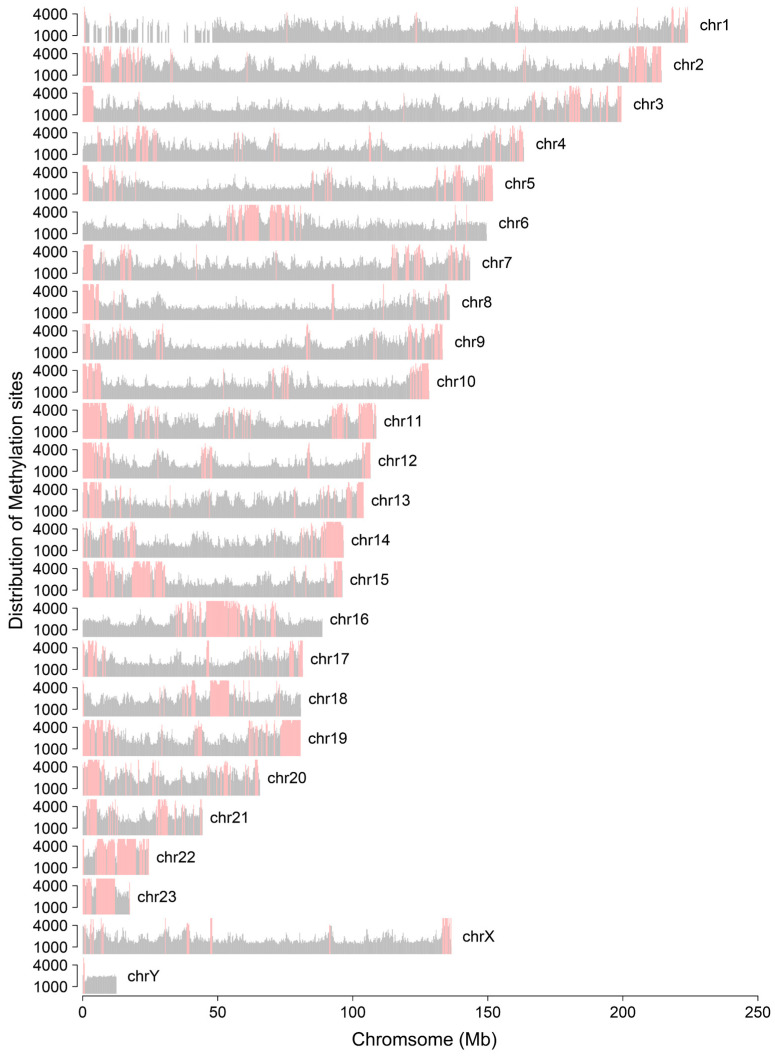
The distribution of methylation sites across all chromosomes. Pink represents hypermethylation and grey represents hypomethylation.

Using a 250 kb sliding window, we visualized the distribution of 5 mc methylation sites across the chromosomes and observed distinct methylation patterns (Figure A15). For instance, hypermethylation patterns at both ends of certain chromosomes, such as chromosomes 2 and 3, may be linked to telomeric regions. Pearson correlation analysis revealed that LINE sequence length was negatively correlated with methylation levels (*r* = −0.6628, *p* < 0.05), while SINE sequence length showed a positive correlation (*r* = 0.5509, *p* < 0.05) (Figure 5B). These findings suggest that LINE sequences are associated with reduced methylation, whereas SINE sequences tend to be linked with increased methylation.

We identified 1,070,617 methylated sites in LINEs and 1,538,439 methylated sites in SINEs, accounting for 19.7% and 26.3% of the total methylated sites, respectively. These sites span 64.6% of LINE sequences and 62.2% of SINE sequences, indicating that a large proportion of LINE and SINE sequences in the *A. albiventris* genome are methylated (Figure 5C). These results demonstrate that the single-base 5 mc methylation maps offer a reliable means to assess genome-wide DNA methylation levels in LINEs and SINEs. Moreover, we found that the methylation levels of both LINEs and SINEs increase with sequence length (*r* > 0.5809) (Figure 5D). Interestingly, the methylation levels in LINEs and SINEs near gene regions were similar to those in distal intergenic regions, showing minimal variation in methylation depending on genomic location (Figure 5E). Additionally, we analyzed the relationship between methylation variability and the evolutionary age of LINE and SINE subfamilies. Our results showed a significant trend of increasing methylation from older to younger elements (*p* < 0.05), with Pearson correlation coefficients of −0.4767 for LINEs and −0.5991 for SINEs (Figure 5F). This suggests that younger LINE and SINE elements tend to have higher methylation levels than their older counterparts, highlighting the evolutionary dynamics of methylation in these retrotransposons.

### 3.6. LINEs and SINEs Distributions Correlate with Global Compartmentalization

**Figure 6:**
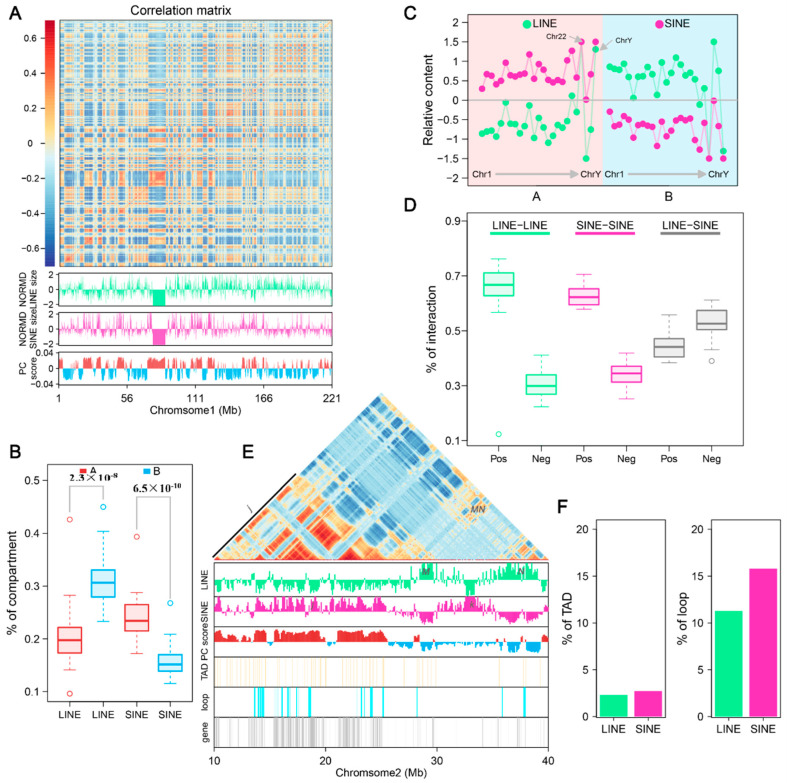
LINE and SINE-rich genomic regions and their association with 3D genome structure: (**A**) Heatmap of normalized interaction frequencies at 100 kb resolution on chromosome 1. Below the heatmap, tracks depict the genomic distribution and densities of LINEs (green) and SINEs (pink), as well as the eigenvalues of the Hi-C matrix, delineating A (positive, red) and B (negative, blue) compartments. In the figure, green represents LINE and pink represents SINE, red represents compartment A, and blue represents compartment B. (**B**) Boxplots comparing the proportion of LINEs and SINEs in A and B compartments. The statistical significance between compartments is annotated above the plots, highlighting compartment-specific enrichment. (**C**) Relative content of LINE and SINE repeats across A and B compartments in different chromosomes. Variations in repeat densities are visualized, with chromosomes partitioned by their compartmental organization. (**D**) Boxplots illustrating the frequency of chromatin interactions between LINE-LINE, SINE-SINE, and LINE-SINE pairs. (**E**) Zoomed-in view of interaction matrix for the genomic region from 10 to 30 Mb on chr2. Below the heatmap: genomic distributions of LINEs, SINEs, A/B compartments, TADs, and loops. LINE-rich regions are labeled as M and N (uppercase), and SINE-rich regions as *j* and *k* (lowercase). In the figure, green represents LINE and pink represents SINE, bright red represents compartment A, blue represents compartment B, light yellow represents TAD, light blue represents loop, and gray represents gene. (**F**) Proportion of TADs and loops in LINE and SINE-rich regions.

**Figure A16:**
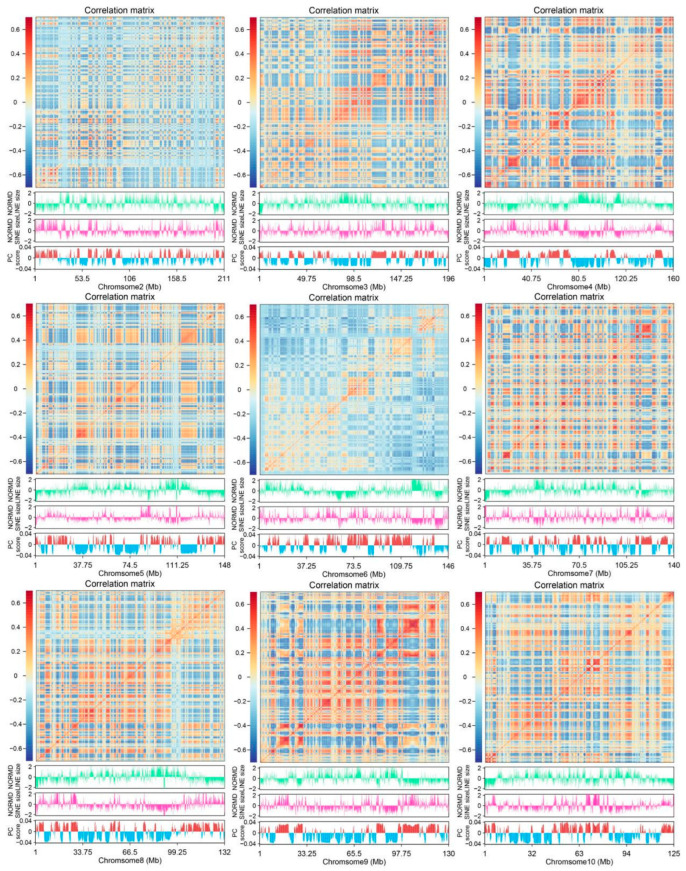

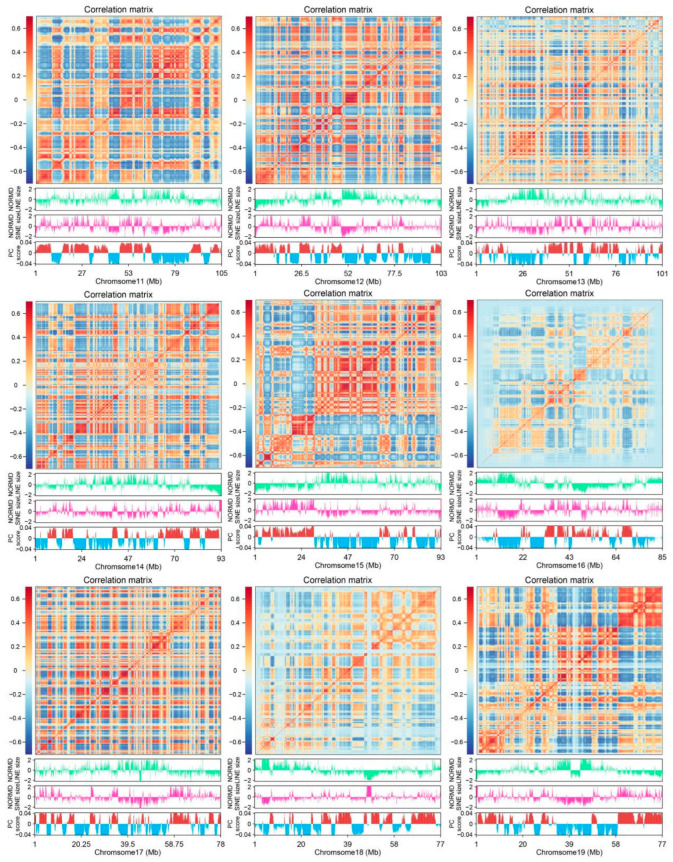

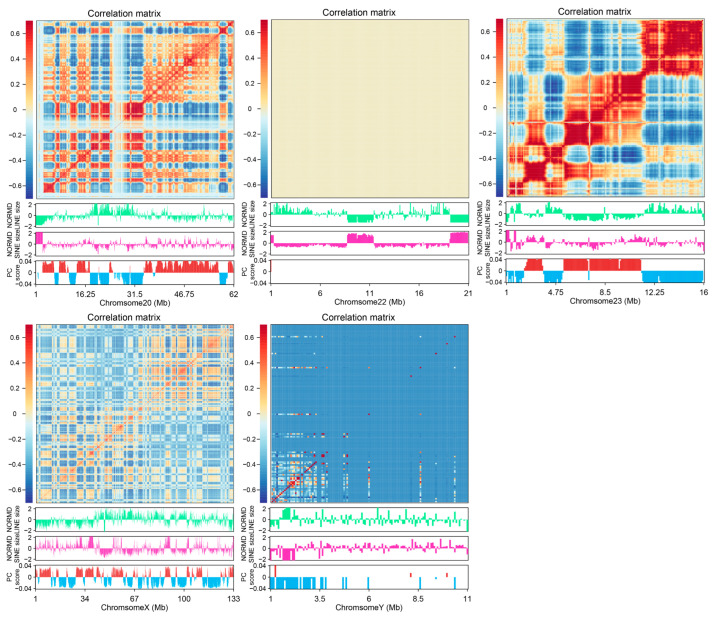
Heatmap of normalized interaction frequencies at 100 kb resolution for each chromosome. Under the heatmap, we show genomic distributions and densities of LINEs and SINEs and eigenvalues of the Hi-C matrix representing A/B compartments. In the figure, green represents LINE and pink represents SINE, red represents compartment A, and blue represents compartment B.

**Figure A17:**
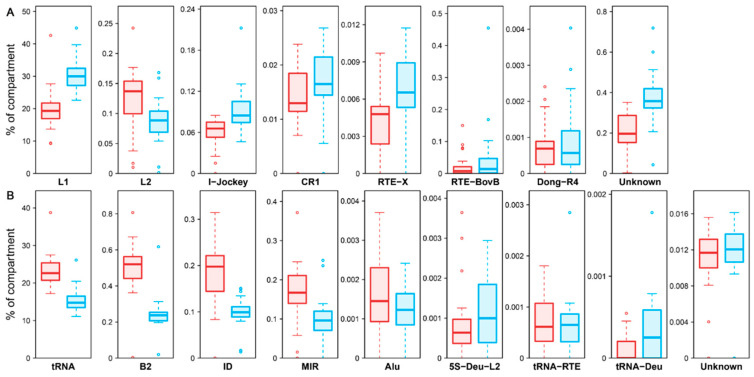
Boxplots showing LINE (**A**) and SINE (**B**) family’s content in A and B compartments. The light red color indicated A compartment, and the light blue indicated B compartment.

The Hi-C technique provides a detailed view of 3D chromatin organization by quantifying interaction frequencies between genomic regions. To investigate the relationship between the 3D genome structure and retrotransposons in the *A. albiventris* genome, we analyzed a published dataset of higher-order chromatin interactions from spleen tissues [3]. Principal component analysis (PCA) of the distance-normalized interaction matrix identified active A compartments and inactive B compartments, which account for 49.99% and 50.01% of the genome, respectively (Figure 6A and Figure A16). Quantitative analysis revealed that SINEs are predominantly found in the A compartment, while LINEs are enriched in B compartments across the genome (Figure 6B and Figure A16), a pattern that is consistent across most chromosomes except for chromosomes 22 and Y (Figure 6C). Certain repeat families, such as RTE within SINEs and LINE2 within LINEs, deviated slightly from this trend, though they represent a small fraction of the genome (Figure A17).

When examining the interaction relationships among retrotransposons using the Hi-C correlation matrix, we observed that interactions between retrotransposon elements of the same type occurred significantly more frequently (*r* > 0) than interactions between different types (*r* < 0) (Figure 6D). Positive correlations between LINEs made up 66.75% of all LINE-LINE interactions, while LINE-SINE interactions showed a slightly lower frequency of positive correlations (47.39%).

To further explore retrotransposon interaction and distribution within the chromatin structure, we focused on a 40 Mb region of chromosome 2 (chr2). Overlaying LINE and SINE features onto the Hi-C correlation matrix revealed distinct patterns of positive and negative interaction blocks, corresponding to alternating LINE-rich and SINE-rich regions (Figure A16). In this region of chr2, two LINE-rich regions (*M* and *N*) in the B compartment and two SINE-rich regions (*j* and *k*) in the A compartment were identified (Figure 6E). Strong interactions were detected between the LINE-rich regions (*MN*) within the B compartment and among SINE-rich regions (*j*) in the A compartment. Minimal interactions were observed between the LINE and SINE-rich regions. Interestingly, one SINE-rich region (*k*) in the B compartment showed negligible interactions with other regions. We also noted that LINE- or SINE-rich regions often span adjacent topologically associating domains (TADs), a trend more pronounced in the enlarged sections of chromosome 2 between 10 and 40 Mb (Figure 6E). TAD and retrotransposon overlap analysis showed that the proportion of TADs in LINE- and SINE-rich regions is nearly identical across the genome (Figure 6F). However, the proportion of chromatin loops in SINE-rich regions was observed to be higher compared to LINE-rich regions, suggesting distinct organizational roles for SINEs within the 3D genome structure.

### 3.7. LINEs and SINEs Are Associated with Spine Formation in A. albiventris

**Figure 7:**
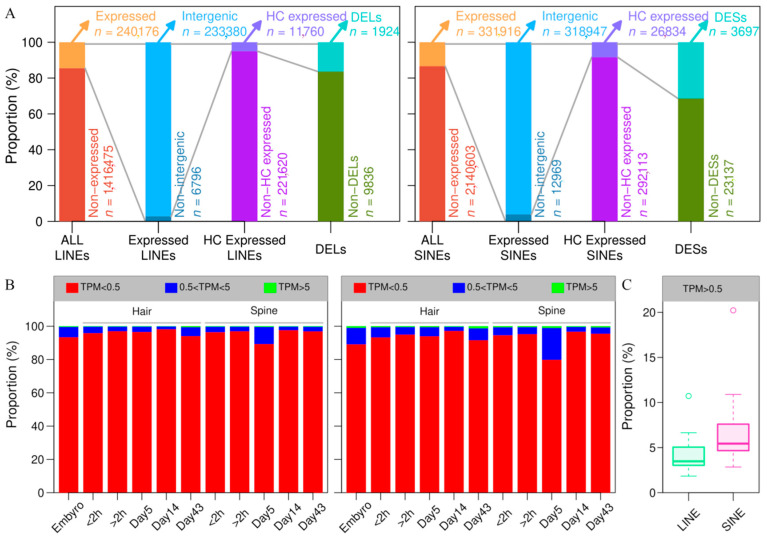
LINEs and SINEs involved in skin development in *A. albiventris*: (**A**) Proportion of LINEs, SINEs, and genes in each category for the corresponding analysis. These include all transposable elements, expressed LINEs and SINEs, and differentially expressed LINEs (DELs) and SINEs (DESs). (**B**) The histogram of different ranges of TPM values. On the left is LINE and on the right is SINE. (**C**) The boxplot of LINE and SINE expression TPM values (greater than 0.5).

To characterize the landscape of LINE and SINE expression during spine development in *A. albiventris*, we applied REdiscoverTE to analyze 22 RNA-seq samples, including 2 embryonic samples, 10 from dorsal skin tissues, and 10 from abdominal skin tissues across three developmental stages. A total of 240,176 LINEs and 331,916 SINEs were identified as being expressed in at least one sample (Figure 7A). To assess data quality, we analyzed the distribution of retrotransposons across various TPM value ranges relative to the total number of expressed retrotransposons (Figure 7B). Similar expression patterns of LINEs and SINEs across FPKM intervals were observed in both abdominal hair and dorsal spine tissues. Throughout all developmental stages, the majority of LINEs and SINEs displayed low expression levels (TPM < 0.5) in both tissue types. A small proportion of these elements exhibited moderate expression (0.5 ≤ TPM < 5), with only a few reaching high expression levels (TPM ≥ 5). Moreover, the proportion of expressed SINEs with a TPM value exceeding 0.5 was significantly higher than that of LINEs (Figure 7C). The expressed LINEs are predominantly located in intergenic regions (96.09%), followed by introns (3.86%) and exons (0.05%), while expressed SINEs are similarly distributed with 97.17% in intergenic regions, 2.80% in introns, and 0.03% in exons. To reduce false positives in the TE expression quantification process, we applied the *filterByExpr* function in edgeR to identify high-confidence (HC) expressed LINEs and SINEs in intergenic regions, yielding 11,760 LINEs and 26,834 SINEs for subsequent analysis, as these are more likely to be actively transcribed (Figure 7A).

**Figure A18:**
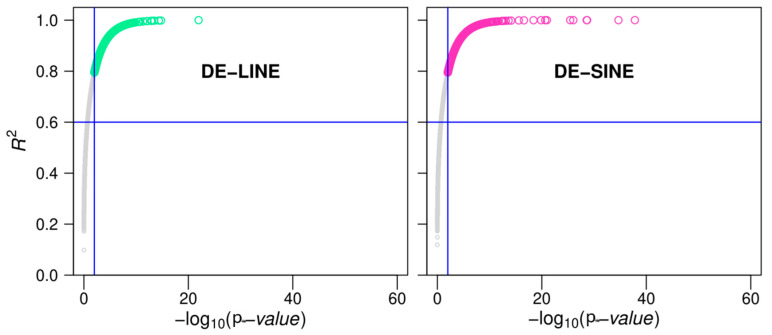
Scatter plot of *R*^2^ vs. −log 10 of the *p*-values depicting differential LINE and SINE expression between abdomen hair and dorsal spine tissues. The blue line represents a standard baseline.

**Figure A19:**
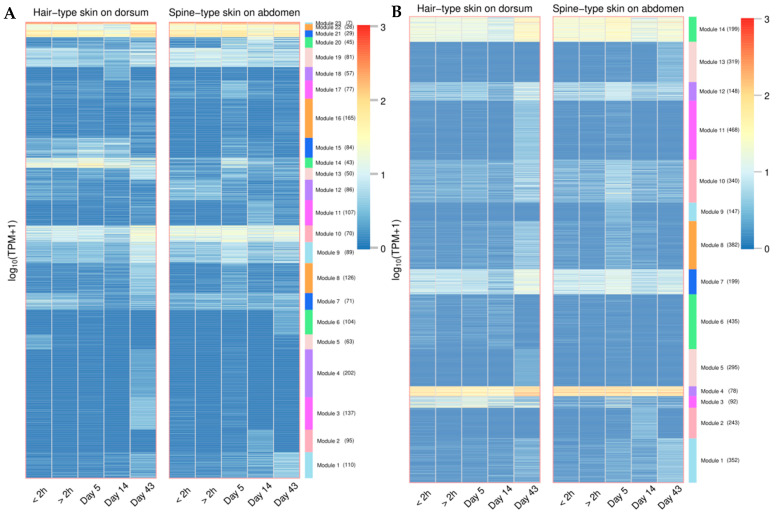
Heatmap of hierarchical clustering analysis for differentially expressed LINEs (DELs) (**A**) and SINEs (DESs) (**B**) in hair-type skin on the dorsum and spine-type skin on the abdomen across different developmental stages. Modules are represented by different colored bars and numbered according to clustering results.

A time-series differential expression analysis between abdomen hair and dorsal spine tissues identified 1924 differentially expressed LINEs (DELs) and 3697 differentially expressed SINEs (DESs) (Figure A18). We performed hierarchical clustering of DELs and DESs in the two tissues, with the optimal number of clusters determined using the silhouette method. The expression patterns of DELs and DESs were categorized into 23 and 14 clusters, respectively (Figure A19). Some clusters show high expression in both tissues, though the expression levels differ between them. For instance, modules 21, 22, and 23 in DELs and modules 4 and 14 in DESs. Interestingly, module 14 in DELs (containing 43 LINEs) and module 3 in DESs (containing 92 SINEs) exhibited high expression in abdominal hair skin tissue during the first stage while showing low expression in other stages. This suggests that these specific LINEs and SINEs may play a role in tissue-specific gene regulation during early spine development.

We further investigated the specific LINEs and SINEs associated with differentially expressed genes (DEGs) located more than 5 kb away, within module 14 of DELs and module 3 of DESs (Table 2). In module 14 of DELs, we identified nine LINEs located in close proximity to differentially expressed genes (DEGs), three of which have functional annotations: AA_009405.1, AA_026619.1, and AA_028322.1. AA_026619.1, annotated as DSG4, encodes a protein that plays a crucial role in cell adhesion within the skin, contributing to the integrity and stability of the epidermis. The correlation coefficients of these LINEs with their adjacent DEGs (>5 kb) ranged from 0.58 to 0.78, except for LINE_421289 and LINE_535022, which exhibited weak and strong negative correlations, respectively. Similarly, module 3 of DESs was found to contain 18 SINEs in close proximity to DEGs such as GPCR5D and AZGP1. The correlation coefficients for 15 of these SINEs and their adjacent DEGs ranged from 0.20 to 0.99, while 3 SINEs showed negative correlations, with SINE_1866950 exhibiting a particularly strong negative correlation of −0.51 with its adjacent gene. These findings underscore the potential involvement of specific LINEs and SINEs in the regulation of genes related to spine development, suggesting that their transcriptional activity at certain stages may influence the development of this unique morphological trait in *A. albiventris*.

## 4. Discussion

Repetitive sequences are a major constituent of many eukaryotic genomics and play an important role in genomic structure, stability, rearrangements, and gene regulation. Moreover, the content of repetitive sequences is positively correlated with genome size [69,70]. In most mammals, repetitive sequences can constitute nearly half of the genome. Among these, retroelement sequences are the most abundant, with many copies of LINE and SINE [21]. In the previous study, mobilome annotation in two hedgehog species revealed that repeats accounted for approximately 58% and 57% of the *A. albiventris* and *E. europaeus* genomes, respectively. This is significantly higher than humans (45%), mice (38%), and other mammals [3,71], showing a tendency for expansion in the hedgehog genome. In the present study, the classification analysis of repeat sequences of the *A. albiventris* genome suggests that this expansion was primarily driven by an increase in SINE content. This pattern was observed in only a few other mammal mobilomes, including those of tree shrews and rabbits [4]. The observation that the number of SINE subfamilies identified through de novo predictions exceed those from homologous annotation suggests that the SINEs of *A. albiventris* have different sequence characteristics compared to other mammals and may undergo distinct evolutionary histories.

In the *A. albiventris* genome, the high proportion of tRNA-derived SINEs is noteworthy, indicating that fewer lineage-specific SINEs contribute to genome expansion compared to the human and mouse genomes. Regarding chromosomal distribution, LINEs and SINEs do not exhibit a strong bias towards specific locations in the *A. albiventris* genome. However, SINEs tend to form denser clusters, with a higher proportion of SINEs located within 0–200 bp intervals compared to LINEs. This clustering of SINEs may be linked to the expression of nearby genes or facilitate recombination and deletion events, though further experimental validation is required to confirm these potential roles. These observations highlight the unique role that repetitive elements, particularly SINEs, play in shaping the genome of *A. albiventris*. In this study, B2 and ID elements, although derived from tRNA genes, were classified separately due to potential differences in sequence features and transposition mechanisms. Given the limited homologous data available for hedgehogs, we relied on RepeatMasker for annotation, classifying them as tRNA-SINEs, though they might represent hedgehog-specific SINEs.

Despite employing a comprehensive approach combining homology alignment and de novo prediction for the classification and annotation of LINEs and SINEs in the *A. albiventris* genome, a subset of consensus sequences remained unassigned. Most of the unannotated LINE sequences clustered together, showing high sequence similarity, suggesting they may belong to an *A. albiventris*-specific LINE superfamily. Additionally, we identified two unknown SINE consensus sequences that could represent a hedgehog-specific SINE family. In hedgehogs, the majority of SINEs are derived from tRNAs, which sets them apart from humans, where Alu elements originate from 7SL RNAs, and mice, where B1 elements are derived solely from 7SL RNAs. However, the mouse genome harbors a wide variety of tRNA-derived SINEs, including B2, B3, B4, MIR, ID, tRNA-Deu, and tRNA-RTE [24,72]. Notably, tRNA-derived SINEs are significantly more abundant than 7SL-derived SINEs in the mouse genome. These SINE families have different genomic distributions, evolutionary ages, and structural features, which contribute to their significant role in chromatin organization, gene expression regulation, and evolutionary processes in mice. This tRNA-derived feature aligns with previous findings in the rabbit genome [4]. The relative age distribution of SINEs in *A. albiventris* suggests that their expansion occurred much earlier, with bursts at approximately 40 Mya and 60 Mya. In contrast, SINE expansion in the rabbit genome took place more recently, consisting mainly of younger lineages [4]. The estimated divergence time between hedgehogs and rabbits, approximately 94 Mya (CI: 91.5–97.4 Mya) [73], predates these SINE expansion events. This indicates that the observed SINE dynamics evolved independently after their divergence, reflecting lineage-specific genomic trajectories. Typically, LINE and SINE superfamilies are classified into distinct families based on consensus sequence similarity, each with varying evolutionary trajectories. These differences can influence genome structure and gene function [13,15].

In the *A. albiventris* genome, LINE1 elements (97.8% of the total LINE content) and tRNA-derived SINEs were classified into six and five families, respectively, each showing unique evolutionary profiles. Among the LINE1 families, two (LINE1A_aAlb and LINE1C_aAlb) exhibited high current activity with numerous copies, while two tRNA-derived SINE families (tRNAA_aAlb and tRNAB_aAlb) also showed significant activity, though their expansion occurred later than that of the two LINE1 families. Active LINEs have the ability to encode proteins that facilitate the reverse transcription and integration of SINEs back into the genome, leading to the co-evolution of LINEs and SINEs, which have dominated ancient mammalian genomes [74]. Interestingly, our analysis revealed that SINEs in the *A. albiventris* genome experienced their major expansion earlier than LINEs, which contradicts the commonly accepted understanding that LINE activity typically precedes SINE bursts. This suggests a unique evolutionary trajectory for retrotransposon expansion in the *A. albiventris* genome. Our definition of “putatively active copies” based on insertion age (<15 Mya) and high copy number has limitations, as it does not account for structural integrity (e.g., presence of intact ORFs) or insertion polymorphism, which are key indicators of activity. For instance, elements with disrupted ORFs may be inactive despite recent insertion. Future studies incorporating these criteria would provide a more robust assessment of transposable element activity.

Similar to most other mammals, LINEs in the *A. albiventris* genome show a tendency to insert into AT-rich, less methylated regions, while SINEs prefer GC-rich, potentially more methylated regions. Our findings also revealed negative correlations between local GC content, methylation levels, and the insertion age of LINEs and SINEs, indicating that older SINE and LINE elements, with lower GC content, may have reduced methylation levels in the *A. albiventris* genome. This pattern is consistent with a previous study that reported lower DNA methylation levels in older Alu/LINE-1 elements compared to younger Alu/LINE-1 in the HapMap LCL GM12878 sample [75]. Additionally, DNA methylation can contribute to genome expansion by depleting CpG sites through TE-mediated deamination and neofunctionalization [76]. Evolutionary analysis of Alu SINEs showed that older Alu elements experienced more CpG loss in their immediate flanks than younger ones [75]. We suspect that SINE expansion in the *A. albiventris* genome may follow a similar mechanism, leading to the generation of new functional elements, as indicated by the decreasing number of methylation sites and the reduction in CpG content over time. In humans and mice, the distribution of B1/Alu and L1 repeats is strongly correlated with A and B chromatin compartments, suggesting their potential contribution to genome folding [30]. A similar pattern was observed in the *A. albiventris* genome. Although we observed that SINE content did not significantly raise the proportion of A compartments, this may suggest a potential role for SINEs in chromatin compartmentalization. However, their expansion alone does not appear to directly drive changes in chromatin structure.

LINE and SINE insertions are frequently found in non-translated regions of genes, such as UTRs and introns, where they can regulate gene expression through different mechanisms [7,21]. SINEs in the UTR regions (including both 5′UTR and 3′UTR) can regulate mRNA stability, alternative splicing, and translation efficiency [77]. Many studies have demonstrated that numerous phenotypic changes in humans and animals are linked to retrotransposon insertions in intron regions [78,79]. In pigs, a 275 bp SINE insertion into the first intron of the *PDIA4* gene was found to be responsible for litter size [80]. Additionally, SINE insertions in long noncoding RNAs (lncRNAs) can regulate the expression of target genes by promoting the translation of overlapping sense protein-coding mRNAs [81]. In the *A. albiventris* genome, our large-scale analysis revealed that LINEs and SINEs, particularly SINEs, with the majority of these elements found in promoter and intron regions. Estécio et al. (2012) demonstrated that SINE B1 elements can influence the activity of nearby promoters, which may contribute to epigenetic reprogramming [82]. In our study, the functional enrichment of genes associated with LINEs and SINEs suggests a potential link to these regulatory mechanisms, though it does not establish direct functional differences. Notably, SINE-associated genes are involved in processes such as chemotaxis, muscle regulation, and skeletal development, which could imply that SINE elements are involved in structural and developmental functions. The expansion of these elements may be associated with the adaptability and evolutionary success of *A. albiventris*, but further studies are needed to clarify the precise role of SINEs in driving phenotypic diversity and environmental responsiveness in this species.

Numerous studies have shown that LINEs and SINEs play crucial roles in gene regulation and genome structure modification [83,84,85]. Many LINE and SINE transcripts have been identified as regulatory RNAs or are involved in forming chimeric transcripts [86]. Hedgehog spines, a unique protective structure made of keratin, are noted for their hardness and sharpness. These spines serve a dual function: providing defense against predators and erecting a sturdy protective barrier in response to threats. Previous research has mapped the transcriptome involved in spine formation in *A. albiventris*, identifying several key candidate genes, such as *SFN*, *Wnt1,* and *KRT1* [48]. A major challenge in understanding TE expression is the accurate quantification of short-read sequences from repetitive regions in the transcriptome. REdiscoverTE addresses this by comprehensively quantifying expression from all repetitive elements, including TEs, in RNA-seq data. One of its key advantages is the ability to specifically model autonomous TE expression [87]. This computational workflow separates reads at the family level based on their genomic location (intronic, exonic, and intergenic), enabling more precise analysis of TE expression dynamics [61]. In this study, we quantified the genome-wide expression levels of LINEs and SINEs using dynamic RNA-seq data from the abdominal and dorsal skin tissues of *A. albiventris*. By using REdiscoverTE to categorize the locations of transposable elements, particularly in intergenic regions, we can infer that these LINEs and SINEs are more likely to be autonomously transcribed in *A. albiventris*.

In this study, we identified differentially expressed SINEs and LINEs in intergenic regions across tissues and developmental stages. Our findings suggest that specific LINEs and SINEs may play a role in tissue-specific gene regulation, particularly during early spine development. Notably, several DELs and DESs were highly expressed in abdominal hair skin tissue during the first developmental stage, indicating that these TEs may be actively transcribed at this stage. Previous work has shown that TEs can act as regulatory elements, influencing the expression of nearby genes through various mechanisms, including enhancer-like activity, modulation of chromatin accessibility, and interaction with transcription factors [12,38,39]. The positive and negative correlations observed in our study between TEs and their adjacent DEGs suggest that these LINEs and SINEs could either promote or suppress the expression of genes critical for spine development. We further identified that a DEL is located near DSG4, a gene that encodes a protein involved in cell adhesion within the skin. We hypothesize that this DEL may be positioned near DSG4, potentially influencing its expression and thereby regulating spine development in *A. albiventris*. Although we screened for potentially actively expressed LINEs and SINEs in intergenic regions using REdiscoverTE, future studies employing techniques such as CAGE-seq, RAMPAGE analysis, melRNA-seq, and strand-specific sequencing are needed to validate active transcription and confirm the regulatory roles of these elements [88,89].

Considering the complexity of TEs and the limitations of short-read sequencing, REdiscoverTE was designed to quantify TE expression at the family or subfamily level, improving quantification accuracy. However, our focus on identifying LINEs and SINEs associated with spine development required quantifying individual elements, which reduces precision for highly repetitive TEs. To mitigate this, we applied stringent filtering criteria, removing many low-expression elements to minimize false positives. We also observed that most of the expressed LINEs were truncated, which complicates the identification of their promoter sequences. Relying solely on sequence characteristics made precise predictions difficult. Thus, future studies using CAGE-seq or full-length sequencing will be crucial for discovering and validating these promoters, and this will be a key focus of our future research. Additionally, the reliance on RepeatMasker for TE annotation and the absence of specific functional annotations may have introduced limitations in identifying certain LINE and SINE sequences, potentially leading to gaps in our dataset. Future studies incorporating long-read sequencing (e.g., PacBio Iso-Seq) and functional annotation approaches will be crucial for improving TE characterization and refining our understanding of their roles in genome evolution and regulation.

## Figures and Tables

**Table 1:** LINE and SINE content in the *A. albiventris* genome.

TE Types	Family	Total Length (Mb)	Percent of the Genome (%)	Copy Number	Number of Cons	De Novo Predicted Cons	Homologous Predicted Cons
LINE	LINE1	709.342	26.768	157,890	320	162	158
	LINE2	3.251	0.123	20,612	38	2	36
	I-Jockey	2.232	0.084	4476	4	3	1
	RTE-BovB	0.934	0.035	1333	4	1	3
	CR1	0.457	0.017	2569	23	0	23
	RTE-X	0.165	0.006	888	3	0	3
	Dong-R4	0.025	0.001	110	1	0	1
	L1-Tx1	0.005	0.0002	24	1	0	1
	Unkown	8.376	0.316	47,737	51	31	20
SINE	tRNA	522.783	19.727	231,903	169	167	2
	B2	10.527	0.397	76,040	3	3	0
	ID	4.176	0.158	36,318	11	10	1
	MIR	4.018	0.152	33,881	19	4	15
	5S	0.062	0.002	1388	2	2	0
	Alu	0.0438	0.002	233	2	1	1
	5S-Deu-L2	0.029	0.001	225	1	0	1
	tRNA-RTE	0.019	0.000749	174	1	0	1
	tRNA-Deu	0.007	0.000252	46	1	0	1
	B4	0.0002	0.0001	3	1	0	1
	Unkown	0.332	0.013	5179	10	4	6

Note: Cons represents consensus sequences.

**Table 2:** Summary of DELs, DESs, and their adjacent differentially expressed genes (DEGs) in *A. albiventris*.

ID	Chr	Start	End	Family	Adjacent DEG	Annotation	*r* ^2^
LINE							
LINE_1120057	chr4	143488317	143488819	L1	AA_009405.1	FAM26E	0.70
LINE_247084	chr11	56863933	56865234	L1	AA_020233.1	-	0.61
LINE_247085	chr11	56867207	56867409	L1	AA_020233.1	-	0.58
LINE_247086	chr11	56867597	56868351	L1	AA_020233.1	-	0.64
LINE_247472	chr11	57402587	57403490	L1	AA_020278.1	-	0.78
LINE_421289	chr14	29764936	29765085	L2	AA_024301.1	-	−0.04
LINE_486246	chr15	48668411	48668813	L1	AA_026619.1	DSG4	0.81
LINE_535022	chr16	35077514	35078004	L2	AA_027161.1	-	−0.20
LINE_552051	chr16	66158943	66159959	L1	AA_028322.1	NLRP10	0.77
SINE							
SINE_1151806	chr2	62824431	62824620	tRNA	AA_003621.1	CDH3	0.20
SINE_1727795	chr4	143485902	143486092	tRNA	AA_009405.1	FAM26E	0.47
SINE_1727797	chr4	143487233	143487444	tRNA	AA_009405.1	FAM26E	0.63
SINE_1866950	chr5	144747966	144748260	tRNA	AA_010439.1	-	−0.51
SINE_1916990	chr6	53175805	53176119	tRNA	AA_010921.1	-	0.91
SINE_1933630	chr6	71455240	71455642	tRNA	AA_011248.1	-	0.96
SINE_1997950	chr7	3354262	3354512	tRNA	AA_012513.1	-	0.29
SINE_1997951	chr7	3354611	3354803	tRNA	AA_012513.1	-	0.62
SINE_2047969	chr7	54084191	54084453	tRNA	AA_013182.1	GPRC5D	0.91
SINE_2047970	chr7	54085862	54086112	tRNA	AA_013182.1	GPRC5D	0.91
SINE_289188	chr10	126357358	126357515	tRNA	AA_018661.1	ENC1	0.92
SINE_355210	chr11	56868368	56868560	tRNA	AA_020233.1	-	0.44
SINE_726222	chr15	26599048	26599240	tRNA	AA_026313.1	AZGP1	0.91
SINE_793552	chr16	9695099	9695276	MIR	AA_027002.1	THRSP	0.99
SINE_812613	chr16	35078151	35078359	tRNA	AA_027161.1	-	−0.15
SINE_812614	chr16	35078597	35079172	tRNA	AA_027161.1	-	−0.02
SINE_812672	chr16	35133514	35133879	tRNA	AA_027161.1	-	−0.29
SINE_833011	chr16	54486666	54486802	tRNA	AA_028166.1	LYVE1	−0.54

Note: *r*^2^ represents correlation coefficient. Detailed annotations of adjacent DEG IDs are provided in Table S2.

## Data Availability

The whole genome sequence data reported in this paper have been deposited in the Genome Warehouse at the National Genomics Data Center (NGDC), Beijing Institute of Genomics, China National Center for Bioinformation, under the accession number GWHEQWD00000000, which is publicly accessible at https://ngdc.cncb.ac.cn/gwh/, accessed on 3 March 2023. The transcriptome sequencing data related to spine development in *A. albiventris* were obtained from the NCBI Sequence Read Archive (SRA) under the accession number PRJNA561241. Additionally, the gene annotation files, key intermediate datasets generated during the analyses, and the main analysis workflow scripts are available at https://github.com/SystemBio-Sdut/Ata_TEs, accessed on 21 March 2025.

## Supplementary Materials

The following supporting information can be downloaded at: https://www.mdpi.com/article/10.3390/genes16040397/s1, Table S1: 51 unclassified LINE consensus sequences; Table S2: Detailed annotations of adjacent DEG IDs.

## Appendix A

**Table A1:** Classification of unknown LINE and SINE subfamilies in the *A. albiventris* Genome.

Unknown Subfamilies	TE Type	De Novo	Homologous	Length (bp)	Predicted Classification	Copy Number
UnL-9_aAlb	LINE	-	DR0188083	2610	LINE_N1	147
UnL-5_aAlb		-	DR0187941	6555		424
UnL-3_aAlb		-	DR0187847	6294		492
UnL-2_aAlb		-	DR0187849	5800		778
UnL-1_aAlb		-	DR0188009	5966		778
UnL-11_aAlb		-	DR0188066	3940		31
UnL-7_aAlb		-	DR0187956	6171		230
UnL-4_aAlb		rnd-6_family-1116	-	2894	RTE-BovB	483
UnL-8_aAlb		-	DR0187787	1092		151
UnL-6_aAlb		rnd-6_family-2127	-	2609	RTE-X	338
UnL-10_aAlb		-	DR0187728	2877		40
UnS-1_aAlb	SINE	-	DR0187830	279	SINE_N1	151
UnS-2_aAlb		-	DR0188050	196		123
UnS-3_aAlb		rnd-1_family-337	-	208	SINE_N2	17
UnS-4_aAlb		rnd-1_family-322	-	123	MIR	17
